# Identification and characterization of an atypical Gαs-biased β_2_AR agonist that fails to evoke airway smooth muscle cell tachyphylaxis

**DOI:** 10.1073/pnas.2026668118

**Published:** 2021-12-02

**Authors:** Donghwa Kim, Alina Tokmakova, Lauren K. Lujan, Hannah R. Strzelinski, Nicholas Kim, Maliheh Najari Beidokhti, Marc A. Giulianotti, Amirhossein Mafi, Jung-A A. Woo, Steven S. An, William A. Goddard, Stephen B. Liggett

**Affiliations:** ^a^Department of Medicine, University of South Florida Morsani College of Medicine, Tampa, FL 33612;; ^b^Materials and Process Simulation Center, California Institute of Technology, Pasadena, CA 91125;; ^c^Rutgers Institute for Translational Medicine and Science, New Brunswick, NJ 08901;; ^d^Center for Translational Science, Florida International University, Port St. Lucie, FL 34987;; ^e^Department of Molecular Pharmacology and Physiology, University of South Florida Morsani College of Medicine, Tampa, FL 33612;; ^f^Department of Pharmacology, Rutgers-Robert Wood Johnson Medical School, The State University of New Jersey, Piscataway, NJ 08854

**Keywords:** β-agonist, β-arrestin, Gs, biasing, asthma

## Abstract

G protein–coupled receptors display multifunctional signaling, offering the potential for agonist structures to promote conformational selectivity for biased outputs. For β_2_-adrenergic receptors (β_2_AR), unbiased agonists stabilize conformation(s) that evoke coupling to Gαs (cyclic adenosine monophosphate [cAMP] production/human airway smooth muscle [HASM] cell relaxation) and β-arrestin engagement, the latter acting to quench Gαs signaling, contributing to receptor desensitization/tachyphylaxis. We screened a 40-million-compound scaffold ranking library, revealing unanticipated agonists with dihydroimidazolyl-butyl-cyclic urea scaffolds. The *S*-stereoisomer of compound C1 shows no detectable β-arrestin engagement/signaling by four methods. However, C1-*S* retained Gαs signaling—a divergence of the outputs favorable for treating asthma. Functional studies with two models confirmed the biasing: β_2_AR-mediated cAMP signaling underwent desensitization to the unbiased agonist albuterol but not to C1-*S*, and desensitization of HASM cell relaxation was observed with albuterol but not with C1-*S*. These HASM results indicate biologically pertinent biasing of C1-*S*, in the context of the relevant physiologic response, in the human cell type of interest. Thus, C1-*S* was apparently strongly biased away from β*-*arrestin, in contrast to albuterol and C5-*S*. C1-*S* structural modeling and simulations revealed binding differences compared with unbiased epinephrine at transmembrane (TM) segments 3,5,6,7 and ECL2. C1-*S* (R2 = cyclohexane) was repositioned in the pocket such that it lost a TM6 interaction and gained a TM7 interaction compared with the analogous unbiased C5-*S* (R2 = benzene group), which appears to contribute to C1-*S* biasing away from β-arrestin. Thus, an agnostic large chemical-space library identified agonists with receptor interactions that resulted in relevant signal splitting of β_2_AR actions favorable for treating obstructive lung disease.

## Significance

We sought β_2_AR agonists for treating obstructive lung diseases such as asthma, in which this receptor relaxes airway smooth muscle (ASM) cells and opens airways. Agonists favoring Gs coupling (leads to airway relaxation) compared with activating β-arrestin (limits effectiveness due to receptor desensitization) were pursued in a 40-million-compound screening library. Of several agonists identified, one was apparently biased away from β-arrestin. Agonist–receptor–G protein modeling revealed different receptor interactions compared with other agonists. The favorable effects of the apparent biasing with this agonist were demonstrated in a physiologic system (ASM relaxation). These studies point to a different structural class of β-agonists that might be used to treat obstructive lung diseases without the adverse effects associated with tachyphylaxis.

Most G protein–coupled receptors (GPCRs) are now recognized as multisignal transducers (1, 2). Early concepts of agonist–receptor interactions were based on the idea that there was a single “active” receptor conformation induced by the binding of any agonist, resulting in an interaction with the heterotrimeric G protein and a universal, singular signal. Generally, the α-subunit of the G protein, upon its dissociation, was considered the primary activator (or inhibitor) of the effector, resulting in the intracellular signal. Subsequently, it became clear that multiple signaling outcomes from activation of a given GPCR can occur from a single agonist due to specific molecular determinants of the receptor triggering independent mechanisms (3–5). As these multiple functions were being identified, it was apparent that agonists with different structures could act at a given receptor to preferentially activate one signal with minimal engagement of others, a property later termed signal biasing (6–8). Biased agonists, then, could represent important advantages over nonbiased agonists due to this signal selectivity, activating a specified therapeutic pathway while minimally evoking unnecessary or deleterious signaling. The pathway selectivity of biased agonists is thought to be established by the stabilization of specific conformation(s) of the agonist–receptor complex via a set of interactions that differ from those of unbiased (also called balanced) agonists (9–12). While it is conceivable that small modifications of established cognate agonists might yield such specialized signaling, significant deviation from common agonist structures may be necessary to meet this goal (13).

The signals/functions of a given GPCR that might be sought for selective activation are defined by the cell type, disease, and desired final physiologic function. In asthma and chronic obstructive pulmonary disease (COPD), active human airway smooth muscle (HASM) cellular contraction limits airflow, representing a major cause of morbidity and mortality. β_2_-adrenergic receptors (β_2_ARs) expressed on HASM cells are the targets for binding of therapeutically administered β-agonists, which relax the cells via a cyclic adenosine monophosphate–mediated mechanism (14). β-agonists are used for treating acute bronchospasm as well as for long-term prevention. However, the HASM bronchodilator response to acute β-agonist is attenuated by receptor desensitization (15), with typical treatments of humans, or isolated HASM cells, leading to a loss of receptor function over time (16–18), clinically termed tachyphylaxis.

Agonist-promoted desensitization of β_2_AR (and other GPCRs) is due to partial uncoupling of the receptor to the G protein, which is initiated by phosphorylation of intracellular Ser/Thr residues of the receptor by G protein–coupled receptor kinases (GRKs) (19, 20). The GRK-phosphorylated β_2_AR recruits β-arrestin1 or β-arrestin2 to these receptors, with subsequent interactions that appear to compete with the receptor for its binding to the Gα subunit, thus attenuating the intracellular response (11, 21). Such competition has been strongly inferred for the β_2_AR (22, 23) and is compelling for rhodopsin–arrestin interactions (24). In addition, β-arrestin binding to GPCRs can initiate receptor internalization and other events such as receptor activation of ERK1/2 (25) through its multiprotein adapter functions. Thus β-arrestin engagement can be considered an early “second signal” of the β_2_AR as well as a desensitization initiator for attenuating the Gs signal. An agonist that is biased toward Gαs coupling (cAMP production and airway smooth muscle [ASM] relaxation) and away from β-arrestin binding (desensitization) would be desirable in treating obstructive lung diseases, since efficacy would not be attenuated acutely, nor would tachyphylaxis be experienced from extended treatment. While biased agonists favoring either G protein or β-arrestin (6) signaling have been described for some GPCRs (such as μ-opioid and type 1 angiotensin II receptors), Gαs biasing has not been apparent from most studies with catecholamine-like compounds for the β_2_AR. Thus, we have little information as to whether the two β_2_AR pathways can be differentially activated in a selective manner by an efficacious agonist, nor is it apparent from a structural standpoint what strategy might be employed to design agonists biased in this manner for this receptor.

In order to find this type of biasing for the β_2_AR, we screened a 40-million-compound scaffold ranking (SR) library that was agnostic to known β_2_AR agonist structures. We found a scaffold in which substitutions of certain R groups led to individual compounds that are apparently Gαs-biased agonists for β_2_AR with no apparent engagement of β-arrestin in model systems. Additional studies in HASM cells revealed a lack of tachyphylaxis of the relaxation effect by the lead compound compared with the most widely utilized β_2_AR agonist, albuterol. The structure of this biased agonist is very different from that of catecholamine-like agonists. To ascertain the mechanism that may underlie this biased activity, we used structural modeling and molecular simulations and studied homologous compounds with different R groups and receptor mutagenesis to predict the interaction sites with the activated β_2_AR. Such studies uncovered distinct structural characteristics that may be responsible for the biasing effect.

## Results

### Screening an SR Library Yields a Scaffold Structure with Compounds that Activate β_2_ARs.

The screening for unique agonists acting at β_2_AR was performed using nontransfected CHW-1102 cells (CHW), which do not endogenously express the receptor, and the same cell line stably transfected with human β_2_AR complementary DNA (cDNA), expressing ∼2,000 fmol/mg of receptor protein (termed CHW-β2). The *a priori* criteria for a compound (or mixture of compounds) to qualify for further studies were the following: 1) statistically significant stimulation of cAMP in CHW-β2 cells over baseline, 2) no stimulation of cAMP from nontransfected CHW cells, 3) a cAMP response from a compound (or mixture of compounds) that was blocked by the βAR antagonist propranolol, 4) a stereoisomer of a single compound which showed a reduced cAMP response compared with the parent stereoisomer, 5) concentration-response data for cAMP that could be fit to a four-parameter least-squares regression equation (sigmoid curve with *R*^2^ > 0.9), and 6) an average Hill coefficient of the fitted curves for a compound being between 0.8 and 1.3.

**Fig. 1.**
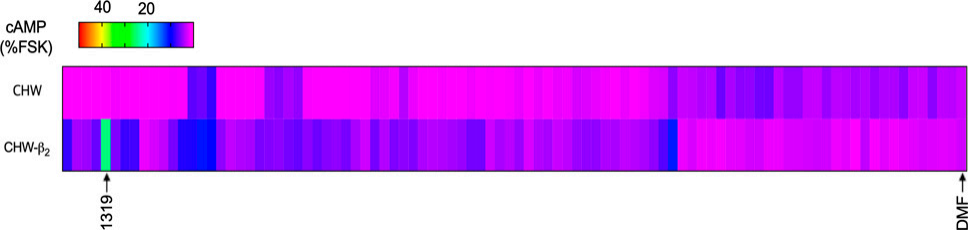
cAMP screening of an SR library of 40 million compounds identifies a dihydroimidazolyl-butyl-cyclic urea scaffold containing β_2_AR agonists. A total of 116 wells each containing mixtures of compounds (250 μg/mL) systematically arranged by scaffold type were used to treat cells transfected to express β_2_AR (CHW-β2 cells, lower row) and nontransfected CHW cells (upper row). The cAMP response for all wells is shown as a heat map indexed to the cAMP response to 10 μM forskolin using the color scheme shown. One well (SR library well 1319) increased cAMP in CHW-β2 cells >sixfold over basal levels (0.25% dimethylformamide [DMF] vehicle). This mixture of compounds did not stimulate cAMP in the nontransfected CHW cells as indicated. Shown are mean results from two sets of determinations.

**Fig. 2.**
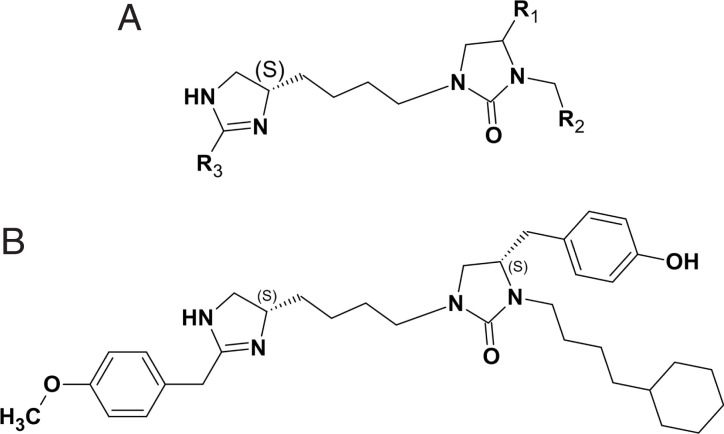
Scaffold structure of compounds in SR library well 1319 and compound C1-*S*. (*A*) Scaffold with the indicated positions of groups (see also *SI Appendix*, Fig. S2) denoted R1, R2, and R3. (*B*) The structure of the individual compound denoted C1-*S* (1 of 12), which was identified from deconvolution of the cAMP responses from the synthesized PS library derived from the SR library sample well 1319.

Our approach for screening 40 million compounds was by utilization of an SR library, in which each well in a sample plate contains thousands of compounds systematically arranged by scaffold type so that each well contains only compounds of a specific scaffold (26). In this case, we studied 87 different SR library sample wells composed of small molecules, cyclic peptides, and linear peptides. From the screening (Fig. 1) of the SR library, a dihydroimidazolyl-butyl-cyclic urea scaffold (Fig. 2*A*), denoted well 1319, increased cAMP over basal by >sixfold in CHW-β2 cells, while there was no significant increase in cAMP in the CHW cells, prompting further study of these compounds. While several other sample wells evoked an increase in cAMP from the CHW-β2 compared with CHW cells (see magenta to blue transitions in Fig. 1), the increase was <1.5-fold, so we concentrated on compounds from well 1319. It is recognized that potential agonists in these other wells, which may be in low concentrations when there are many compounds per well, might be missed in this screening. Nevertheless, the approach has been successful in identifying unique agonists for a number of GPCRs (27, 28), and regardless, agonists in the 1319 well appeared to be suitable for further study based on efficacy.

### Screening of a Positional Scanning Library Yields Individual Atypical β_2_AR Agonists.

In order to predict the relative efficacy of each of these compounds from the well 1319 from the SR library, we employed a computational approach with a custom-built positional scanning (PS) library, as described previously (29, 30). This PS library was used to predict the relative activities of all compounds in the library through the use of exponentially fewer samples. For SR library sample well 1319, we used 116 sample wells to assess the relative efficacy of all 56,610 different dihydroimidazolyl-butyl-cyclic ureas. The 56,610 compounds came from incorporating 34 different functionalities at the R1 position, 37 at the R2 position, and 45 functionalities at the R3 position (34 × 37 × 45 = 56,610). Samples derived from well 1319, termed 1319-1 to 1319-34, contain all the dihydroimidazolyl-butyl-cyclic ureas systematically arranged by their R1 functionality. For example, a given sample well contains all 1,665 dihydroimidazolyl-butyl-cyclic ureas fixed with an *S*-methyl at the R1 position, while another contains all 1,665 compounds with *S-*benzyl fixed at R1. In a similar manner, sample wells were created with fixed R2 or fixed R3 positions.

**Fig. 3.**
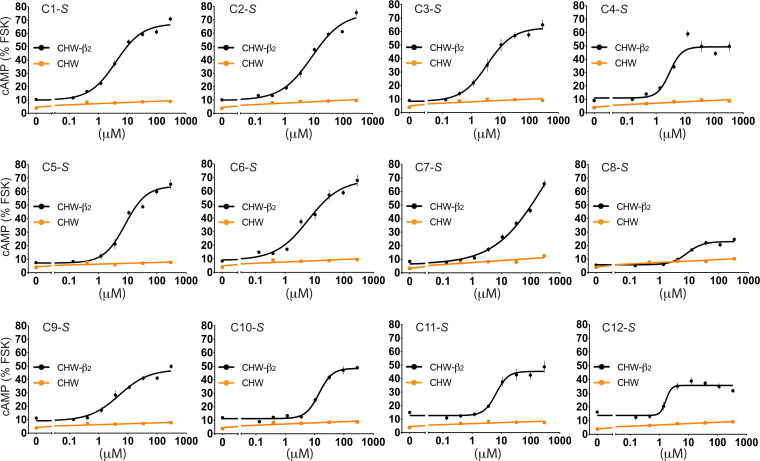
Dose–response curves of cAMP production in CHW-β2 and nontransfected CHW cells from individual compounds denoted C1-*S* through C12-*S* derived from deconvolution of the PS library. Reference *SI Appendix*, Fig. S2 for the structures and Table 1 for a summary of the derived parameters from the curve fits. Results are mean ± SE of four to six experiments.

**Fig. 4.**
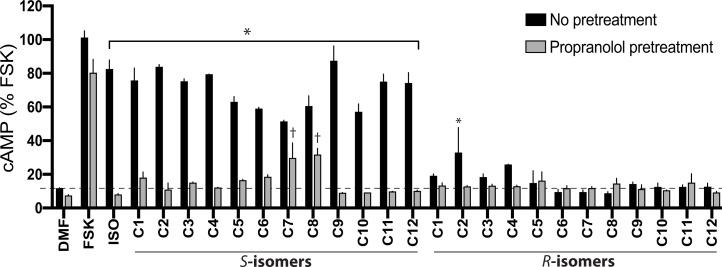
Pharmacologic properties of individual *S-* and *R*-stereoisomer compounds C1 through C12. CHW-β2 cells were pretreated with vehicle or 10 μM of the βAR antagonist propranolol for 5 min. Then the cAMP responses to 50 μM of the individual *S-*active compounds or their *R-*stereoisomers at the R1 position (Fig. 2*A* and *SI Appendix*, Fig. S2) were determined. *, response to a compound (vehicle pretreated, black bars) is greater (*P* < 0.01) than baseline (0.25% dimethylformamide [DMF]); for all gray bars except for C7-*S* and C8-*S*, propranolol pretreatment resulted in no significant (*P* > 0.05) stimulation by a compound over baseline; †, *P* < 0.05 versus baseline. Results are mean ± SE from three experiments.

cAMP screening resulted in several positive sample wells from the PS library, with an example shown in *SI Appendix*, Fig. S1, in which responses to the compounds of the 1319 scaffold are arranged by the groups located at position R1 (Fig. 2*A*). We deconvoluted the PS library screening results by selecting multiple functionalities from each of the R1, R2, and R3 positions and made the combinations of all of them. In order to do this, we first rank-ordered the samples in each of the three positions by cAMP responsiveness and then analyzed the data for trends in structure activity relationships as described (29, 30). Using this approach, 12 *S*-compounds (denoted C1-*S* through C12-*S*) and their *R*-stereoisomers at the R1 position (denoted C1-*R* through C12-*R*, respectively) emerged for synthesis and further study (see structures in *SI Appendix*, Fig. S2). Based on the PS data, the *S*-compounds were predicted to be active, whereas the *R*-compounds were predicted to have significantly less activity, if any. cAMP responses to multiple concentrations of the individual compounds C1-*S* through C12-*S* were determined (Fig. 3), with mean parameters of the individual curve fits shown in Table 1. Each of the *S*-stereoisomers stimulated cAMP to various extents as indicated. Additional cAMP studies were performed with the *S*- and *R*-stereoisomers of C1 through C12 to ascertain stereoselectivity and the response in the presence of the antagonist propranolol (Fig. 4). We noted that the *R*-analogs evoked very little, or no, stimulation of cAMP over baseline (Fig. 4). In addition, most of the agonists exhibited full blockade by propranolol (cAMP level no different from basal), except for C7-*S* and C8-*S*. The C7-*S* response (Fig. 3) could not be fit to a sigmoid curve, and some other *S*-analogs had Hill coefficients outside the prespecified range (Table 1). Based on the lack of full antagonism by propranolol and/or the Hill coefficients, seven *S*-isomer compounds were not studied further.

**Table 1.** Functional characterization of agonists C1-C12 as determined by cAMP production in whole cells

Compound	EC_50_ (μM)	R_min_ (% FSK)	R_max_ (% FSK)	R^2^	Hill coefficient
C1-*S*	4.23 ± 1.00	9.44 ± 0.88	66.98 ± 2.84	0.958 ± 0.005	1.068 ± 0.159
C2-*S*	6.67 ± 2.01	11.76 ± 2.6	66.47 ± 0.86	0.952 ± 0.012	1.210 ± 0.041
C3-*S*	4.31 ± 1.69	8.40 ± 1.40	62.24 ± 6.59	0.949 ± 0.018	1.131 ± 0.076
C4-*S*	3.52 ± 0.52	10.29 ± 4.77	43.53 ± 11.67	0.914 ± 0.024	2.827 ± 0.291
C5-*S*	7.66 ± 0.95	8.16 ± 0.64	68.76 ± 10.30	0.970 ± 0.007	0.948 ± 0.026
C6-*S*	6.53 ± 0.89	8.83 ± 0.92	68.74 ± 6.21	0.896 ± 0.058	0.819 ± 0.150
C7-*S*	>100	3.18 ± 2.27	N/A	N/A	N/A
C8-*S*	8.35 ± 1.50	5.75 ± 1.42	22.87 ± 5.11	0.950 ± 0.010	1.724 ± 0.086
C9-*S*	6.87 ± 0.81	10.37 ± 1.29	47.79 ± 5.28	0.946 ± 0.003	1.648 ± 0.103
C10-*S*	16.72 ± 3.06	11.01 ± 0.63	50.32 ± 3.73	0.956 ± 0.003	1.643 ± 0.052
C11-*S*	5.94 ± 0.46	12.78 ± 3.39	46.44 ± 8.89	0.945 ± 0.017	2.764 ± 0.379
C12-*S*	1.64 ± 0.35	14.28 ± 3.03	35.89 ± 5.25	0.904 ± 0.028	3.588 ± 0.462

CHW cells were stably transfected to express human β_2_AR, and cAMP levels determined after a 10-min exposure to multiple concentrations of the indicated agonists. The EC_50_ for isoproterenol = 2.5 nM, with R_max_-R_min_ values = 52.3. FSK, forskolin; N/A, not applicable due to lack of curve fit.

### Identification of PS Library–Derived β_2_AR Agonists Favoring Gαs Over β-Arrestin.

**Fig. 5.**
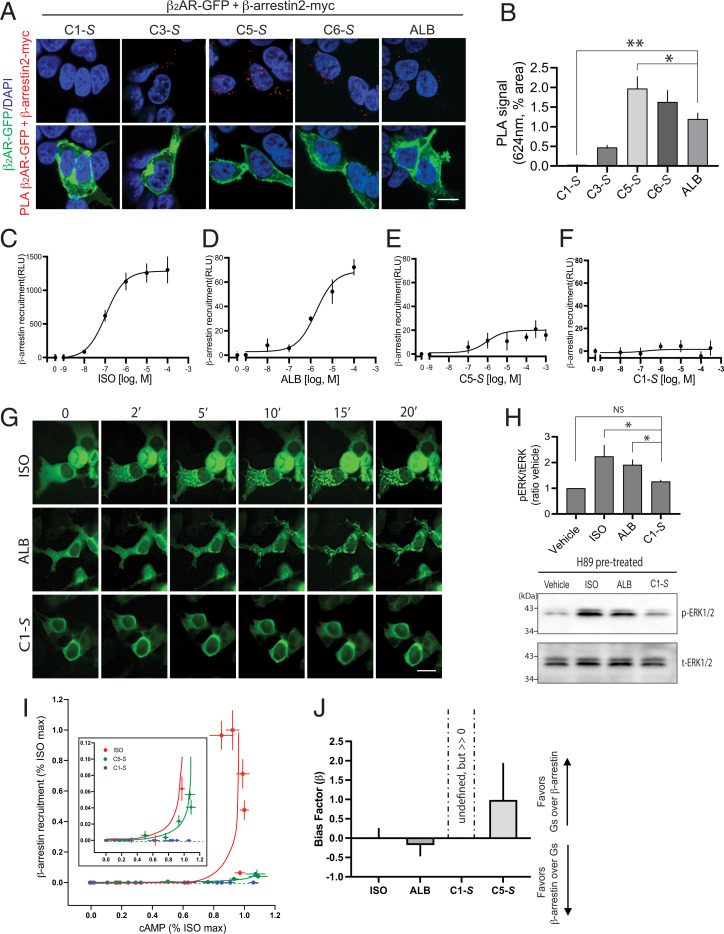
The agonist C1-*S* favors β_2_AR Gαs over β-arrestin interaction. (*A–H*) C1-*S* fails to promote β_2_AR interaction with β-arrestin. (*A*) Representative PLA results from transfected HEK-293 cells treated with the unbiased agonist albuterol (ALB, 10 μM) or the indicated compounds (300 μM). The red signal represents agonist-promoted close proximity of β_2_AR-GFP and β-arrestin2–myc, which was only found in cells that were expressing β_2_AR-GFP (green signals). (*B*) Results from imaging in the red spectra from five independent PLA experiments at the same concentration of agonist as in *A*; *P < 0.05, **P < 0.001; the PLA signal is different from albuterol. Reference *SI Appendix*, Fig. S3 for agonist dose–response results. (*C–F*) β-arrestin2 binding to β_2_AR as determined by the ECA (*n* = 3 to 5). Response curves from isoproterenol (ISO), ALB, and C5-*S* show concentration-dependent increases in the β-arrestin signal (*C–E*), while C1-*S* did not (*F*). (*G*) Images of agonist-promoted recruitment of β-arrestin–GFP to cell-surface puncta by the full βAR agonists ISO (1 μM) and the partial agonist ALB (10 μM) but not the partial agonist C1-*S* (300 μM). Image is representative of four experiments. (*H*) Agonist-dependent, β-arrestin–mediated activation of ERK1/2 is observed with ISO and ALB but not with C1-*S*. The bar graph is from five experiments, and the Western blot shows the Erk1/2 activation observed in a representative experiment. *P < 0.05 versus vehicle control; NS, not significant. (*I*) Bias plot of cAMP versus β-arrestin2 binding for ISO, C1-*S*, and C5-*S* indexed to the maximal ISO responses. Data shown are mean and 95% CIs from three to four experiments. C1-*S*, which showed no detectable β-arrestin2 binding, has a flat curve with nonoverlapping values at most concentrations (see *Inset*). In some cases, the 95% CI bars were smaller than the plotting symbol and are not shown. (*J*) Bias factors (β) for the indicated agonists, using data from the BRET2 assay for Gs coupling and the ECA for β-arrestin binding, were calculated using the formula shown in *SI Appendix*, *SI Expanded Methods*. See also *SI Appendix*, Fig. S5. Since β-arrestin binding was not detected for C1-*S*, the calculated value for β is undefined but is greater than 0. Results are shown as mean ± 95% CIs from four to five experiments. ALB and C5-*S* were not statistically different from 0 (i.e., balanced agonists). (Scale bars in *A* and *G*, 10 μm.)

The remaining S-isomer compounds were further assessed for agonist-promoted β-arrestin binding. We first used a proximity ligation assay (PLA) as previously described (31), transfecting carboxyl-terminal GFP-tagged β_2_AR and carboxyl-terminal myc-tagged β-arrestin2 cDNA expression constructs into human embryonic kidney (HEK)-293T cells. Confocal microscopy reveals a red emission when the two proteins are at least within 30 nm in proximity. Here, we used the β_2_AR-specific partial agonist albuterol as the benchmark agonist, given its widespread use as a bronchodilator for asthma and that the compounds from the screen are also partial agonists. Representative results are shown in Fig. 5*A*, with quantitative results in Fig. 5*B*. Studies using the PLA revealed receptor binding to β-arrestin2 by the agonists C5-*S* and C6-*S* that was comparable to albuterol, with recruitment to a lesser degree by C3-*S*. In contrast, C1-*S* (see structure in Fig. 2*B*) showed no β-arrestin2 binding. Results from dose–response experiments using the PLA with albuterol, the full agonist isoproterenol, and C1-*S* are shown in *SI Appendix*, Fig. S3. Dose-dependent increases in β-arrestin binding were found with albuterol and isoproterenol, but there was no detectable signal for C1-*S* at any concentration tested.

To further explore this C1-*S* phenotype, dose–response experiments were performed using an enzyme complementation assay (ECA, “PathHunter”), which has been shown to be highly sensitive for detection of β-arrestin binding to GPCRs (32). Here, we used Chinese hamster ovary (CHO) cells transfected to express a β-arrestin2 that is fused to a fragment-deficient β-galactosidase and a β_2_AR that is tagged at its carboxyl terminus with the complementation fragment. Upon agonist-promoted binding of β-arrestin to the receptor, β-galactosidase is reconstituted to yield active enzyme, which is detected by luminescence. Using this approach, we detected dose-dependent β-arrestin binding to β_2_AR from isoproterenol and albuterol, with the half-maximal effective concentrations (EC_50_s) of ∼100 nM and ∼1,800 nM, respectively (Fig. 5 *C* and *D*). These results indicate a 1.22 log difference in the potency of the two agonists for promoting β-arrestin binding to β_2_AR, which is similar to the 1.11 log difference previously reported by others using a similar assay (32). Furthermore, the C5-*S* compound was noted to promote β-arrestin binding (Fig. 5*E*), consistent with the PLA. However, there was a lack of a response to C1-*S* in the β-arrestin ECA, with no concentration causing a signal greater than basal (vehicle alone, Fig. 5*F*). Given that the PLA and ECA both showed a lack of β-arrestin binding for C1-*S*, we surmised that this agonist in fact does not evoke a significant β-arrestin response compared with isoproterenol or albuterol within the limitations of these two assays. It has been reported (33) that some agonists at a given GPCR, but not all [e.g., the D2 agonist 75A (33)], display increased β-arrestin binding responsiveness when GRK2 is overexpressed, probably due to an increase in assay sensitivity. We thus performed additional ECA experiments overexpressing GRK2 by >8-fold (*SI Appendix*, Fig. S4*A*) with the C1-*S* agonist. We found no consistent increase in the β-arrestin signal from C1-*S* (R^2^ value for the global best fit = 0.217) under these conditions. Inspection of the change in the luminescence signal with GRK2 overexpression compared with those with endogenous expression at each concentration of C1-*S* showed small positive as well as negative fluctuations in the signal amplitude (*SI Appendix*, Fig. S4*B*), which were not statistically significant, confirming the apparent bias of C1-*S* at β_2_AR even under conditions of GRK overexpression. We also studied C1-*S* using a qualitative method, in which the cellular redistribution of GFP-β-arrestin2 in response to agonist was monitored over time in live cells with fluorescent confocal microscopy, with the expectation that this morphologic event would be absent with C1-*S*. As we and others have described (15, 34), β-arrestin2 recruitment to β_2_AR under these conditions is associated with a change from a homogenous distribution of GFP-β-arrestin2 in the cytosol to punctate accumulations at the cell surface. This response was observed with isoproterenol, and to a lesser extent with albuterol, but not with the C1-*S* agonist (Fig. 5*G*). Finally, it has been established (25) that β_2_ARs activate ERK1/2 via dual independent mechanisms: a cAMP/PKA-Gαs–dependent pathway and a β-arrestin–dependent, Gαs-independent pathway. Thus, cAMP-independent activation via β_2_AR of ERK1/2 acts as a surrogate measure of agonist-promoted β-arrestin binding to the receptor. Thus, we measured ERK1/2 phosphorylation in HASM cells treated with the PKA inhibitor H89 to isolate the β-arrestin component. Isoproterenol and albuterol both activated ERK1/2 under these conditions (Fig. 5*H*), consistent with the previously described agonist-dependent, β-arrestin–mediated event (25). The albuterol signal is less than that of isoproterenol, supporting the less efficacious β-arrestin signaling of albuterol measured by the other methods. In contrast, and in concordance with the aforementioned three other different assays, C1-*S* at a concentration of 300 μM failed to promote ERK1/2 phosphorylation over baseline (Fig. 5*H*). Taken together, these experiments, which used different approaches to ascertain agonist-dependent β-arrestin engagement by β_2_AR, all point to marked apparent biasing of C1-*S* away from β-arrestin signaling.

A bias plot was created using data from the cAMP and the β-arrestin ECA measurements (Fig. 5*I*) for isoproterenol, C1-*S*, and C5-*S*. The isoproterenol curve represents a full, balanced agonist. By inspection of the C1-*S* responses, it is apparent that cAMP production occurs at doses at which β-arrestin recruitment is minimal or not detected (see also insert), distinguishing C1 from isoproterenol as being biased away from β-arrestin engagement. Qualitatively, C5 appears to be balanced for the two responses (Fig. 5*I*). To quantitatively evaluate agonist bias, we calculated the bias factor β using measurements of β-arrestin engagement by the ECA method and receptor–Gs coupling by a bioluminescence resonance energy transfer (BRET2)–based method (35) (“TRUPATH,” see *Methods*). Here, cells were transfected to express β_2_AR, RLuc8-Gαs (energy donor), Gβ, and Gγ-GFP2 (energy acceptor). Agonists display a dose-dependent decrease in BRET2 as the Gs heterotrimer dissociates (35). Although both the cAMP assay and the BRET2 assay are conducted with transfected (overexpressed) cells, the latter shows less system bias (*SI Appendix*, Fig. S5*A*). The transduction coefficients of agonists for β_2_AR signaling to the two pathways were used to calculate β indexed to isoproterenol as the balanced reference agonist by the logistic equiactive method (32, 36) (see *Methods* and *SI Appendix*, *SI Expanded Methods*). Results are summarized in Fig. 5*J* and *SI Appendix*, Fig. S5 *B* and *C*. Using this measure, a balanced (unbiased) agonist has β = 0. When β > 0, Gs signaling is favored compared with β-arrestin signaling, while β < 0 indicates a favoring of β-arrestin signaling compared with Gs signaling. Albuterol was thus found to be balanced (Fig. 5*J*), consistent with previous reports such as that of Rajagopal et al. (32). Since β-arrestin binding was absent with C1-*S* by the PLA and ECA (as well as the confocal recruitment and the ERK1/2 studies), β cannot be defined per se for this agonist (see equation in *SI Appendix*, *SI Expanded Methods*). However, since C1-*S* stimulates cAMP (Fig. 3) and is efficacious in relaxing HASM (see next section), without detectable β-arrestin signaling, this compound appears to display biasing away from β-arrestin while retaining signaling to Gαs, and thus, β is >0. The related compound C5-*S* was found to exhibit balanced signaling (Fig. 5*J*).

### Agonist C1-*S* Fails to Evoke Physiologic Desensitization of β_2_AR-Mediated HASM Cell Relaxation.

**Fig. 6.**
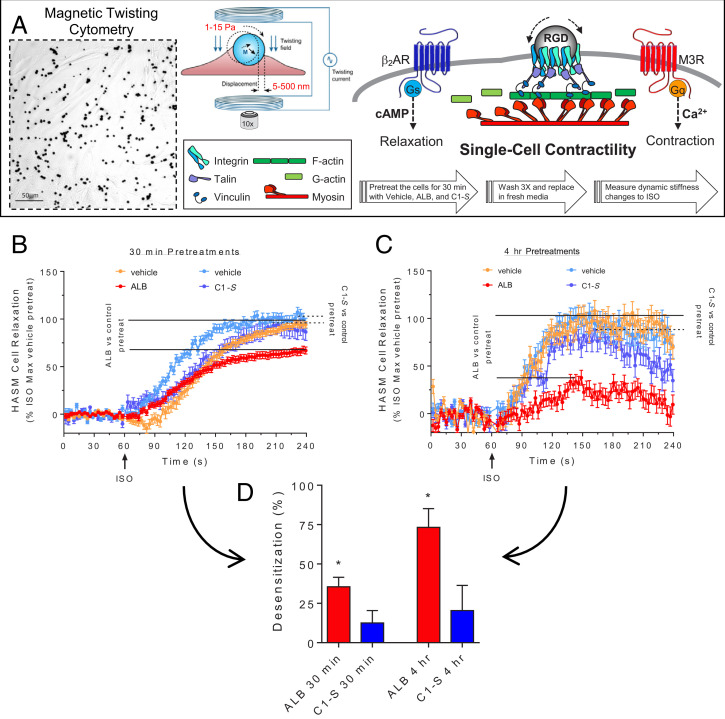
Agonist C1-*S* fails to evoke desensitization of HASM β_2_AR-mediated relaxation. (*A*) Single-cell mechanics of HASM cells were studied using MTC. RGD-coated ferrimagnetic microbeads were attached to integrin receptors. Cells were magnetized horizontally and then twisted in a vertically aligned magnetic field. A decrease in the twisting force was quantified by lateral bead displacement in response to the application of various βAR agonists added to the media. A decrease in stiffness correlates with ASM cell relaxation. The desensitization protocol is shown by the large arrows (see also *Methods*). (*B* and *C*) ALB, but not C1-*S*, preexposure evokes desensitization of β_2_AR-mediated HASM cell relaxation. HASM were pretreated with vehicle (control) or 1.0 μM ALB or 100 μM C1-*S* for 30 min (*B*) or 4 h (*C*) and washed, and then the β_2_AR relaxation response to 10 μM ISO was determined. (*D*) Maximal desensitization to ALB and C1-*S*. ALB caused desensitization of the relaxation response with 30-min and 4-h pretreatment, while C1-*S* evoked no statistically significant desensitization with either pretreatment time. *P < 0.01 versus vehicle pretreatment responses to ISO. Results are from measurements from 103 to 387 cells per condition.

C1-*S* displayed the desired signal biasing (Gs coupling with minimal to no β-arrestin binding) that would potentially translate to the therapeutic action we sought, which is HASM relaxation (activation of cAMP) without desensitization. To verify the lack of C1-*S*–mediated β-arrestin binding in a functional assay of desensitization, β_2_AR transfected cells attached to plates in culture were pretreated with vehicle (control), albuterol, and C1-*S* for 10 min, washed, and then the cells were challenged with isoproterenol and cAMP levels determined after 15 min. As shown in *SI Appendix*, Fig. S6, albuterol pretreatment resulted in a decreased cAMP response to isoproterenol, consistent with short-term agonist-promoted desensitization amounting to ∼68%. In contrast, C1-*S* pretreatment was not associated with a significant loss of β_2_AR function as determined by isoproterenol-promoted cAMP accumulation. To address the physiological consequences of this apparently favorable biasing, magnetic twisting cytometry (MTC) was utilized to quantify changes in the cytoskeletal stiffness of HASM cells. Here, cells were tagged with ferrimagnetic microbeads, and the decrease in cell stiffness (“relaxation”) in response to β-agonist was measured (37, 38) (see Fig. 6*A* and *Methods*). First, we confirmed that C1-*S* relaxes HASM cells as would be expected for a β_2_AR agonist. C1-*S* was found to relax HASM from baseline by ∼35%, confirming efficacy in the physiologic model (*SI Appendix*, Fig. S7). To address agonist-promoted desensitization, we utilized our previously described protocol with these HASM cells (39), which is summarized in Fig. 6*A*. Cells were treated with vehicle, albuterol (positive β-agonist control), and C1-*S* for 30 min and for 4 h. Cells were then washed and subsequently exposed to the full βAR agonist isoproterenol to assess receptor function by real-time measurements of cell relaxation. Albuterol pretreatment for 30 min resulted in a 35 ± 6.0% loss of the relaxation response to isoproterenol (i.e., ∼35% desensitization, Fig. 6 *B* and *D*). In contrast, C1-*S* pretreatment resulted in no statistically significant (12 ± 7.9%) decrease in the subsequent β_2_AR response to isoproterenol (Fig. 6 *B* and *D*). With a 4 h agonist pretreatment, desensitization from albuterol was >70%, while C1-*S* exposed cells responded to isoproterenol with no significant degree of desensitization (Fig. 6 *C* and *D*). These results confirm the biased agonist phenotype of C1-*S* using a physiologically relevant function, in the target cell of interest, with endogenous expression of receptor, GRKs, and β-arrestins.

### The *R*-stereoisomer of C1 Blocks Agonist Activation of β_2_AR.

Although the potency of C1-*S* was somewhat low, we nevertheless expected that C1-*S*, and perhaps C1-*R*, would compete with ^125^I-iodocyanopindolol (^125^ICYP) (40) to some extent at the binding pocket. As shown in *SI Appendix*, Fig. S8, C1-*S* and C1*-R* both competed for ^125^ICYP at the membrane-expressed β_2_AR. The C1-*R* compound, which did not stimulate cAMP over baseline, competed with ^125^ICYP with a K*_i_* that was ∼10-fold higher than C1-*S* (*SI Appendix*, Fig. S8). These radioligand binding competition results and the functional results shown in Fig. 4 suggest that C1-*R* does in fact bind to the receptor but fails to activate it, implying that it could act as an antagonist. To further address this possibility, we studied cAMP stimulation in CHW-β2 cells by the agonist albuterol at its EC_50_ in the absence and presence of C1-*R* to ascertain if binding of C1-*R* was sufficient to block the access of albuterol to the receptor binding sites that are required for receptor–Gαs coupling. These studies showed a dose-dependent blockade of the albuterol cAMP response, with the highest concentration of C1-*R* causing complete blockade comparable to that of the antagonist propranolol (*SI Appendix*, Fig. S9).

### Predicted Binding Sites of C1-*S*, C1-*R*, and C5-*S* to the Activated State of β_2_AR (Σ_act_).

To understand the structural basis for C1-*S* agonism versus C1-*R*, we carried out a series of molecular dynamics (MD) studies that build upon our progress in predicting the structures of the agonist-bound states of the μ-opioid receptor (μ-OR) (11), δ-opioid receptor (δ-OR) (41), and adenosine receptor subtypes (42, 43). For the current work, we started with the activated structure for the unbiased agonist BI167107 complexed with β_2_AR-Gs as defined by its crystal structure (Protein Data Bank [PDB] 3SN6) (44), then removed the agonist, keeping only the receptor for initial docking of the C1-*S* and C1-*R* structures. We then reconstructed the complex of the receptor with anchored G protein. The predicted structures of C1-*S* and C1-*R* docked to β_2_AR-Gs were optimized assuming that the imidazole functionality is protonated. We used partial charges from quantum mechanics (QM) (the B3LYP flavor of Density Functional Theory) combined with the 6-31G** basis set using Jaguar (from Schrödinger).

**Fig. 7.**
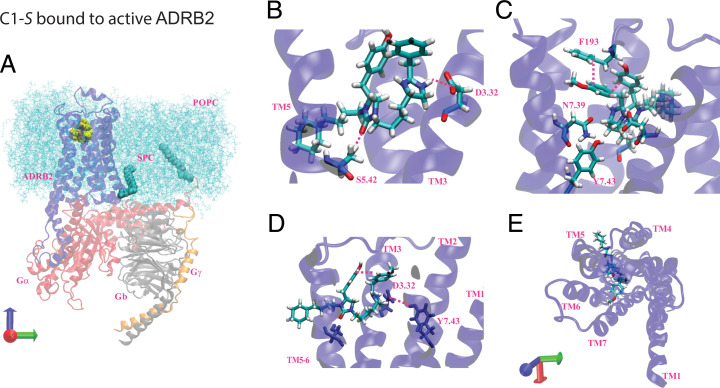
Predicted binding site for C1-*S* binding to β_2_AR coupled to Gs in explicit membrane and water. (*A*) The minimized activated β_2_AR (blue)-C1-*S* (yellow)-Gs protein (Gαs [red], Gβ [gray], and Gγ [orange]) in the phosphatidylcholine (POPC) membrane (light blue). (*B–D*) Selected interactions of C1-*S* with activated β_2_AR: (*B*) the imidazole forms an SB to Asp113^3.32^ and an HB to Asn312^7.39^, (*C*) the urea forms an HB to Ser203^5.42^, and π–π stacking of both aromatic rings of the agonist to each other and to Phe193^ECL2^ is evident, (*D*) internal receptor interaction of Asp113^3.32^ with Tyr316^7.43^, (*E*) upper view of the compound in the TM pocket.

**Fig. 8.**
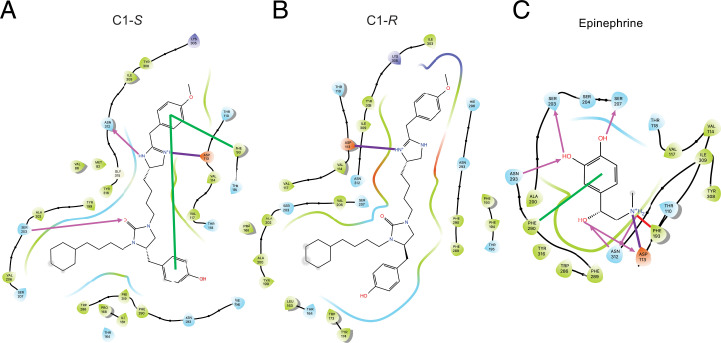
Comparison of predicted binding site pharmacophores for three ligands bound to active-state β_2_AR. (*A*) C1-*S* binding sites include HB (pink arrows) to Ser203^5.42^ and Asn312^7.39^; SB (purple line) to Asp113^3.32^; and Pi–Pi stacking at Phe193^ECL2^ with an internal aromatic bond (green line). (*B*) The binding site of C1-*R* is characterized primarily by the SB to Asp113^3.32^. (*C*) Epinephrine binding sites include HB to Ser203^5.42^, Ser207^5.46^, Asn293^6.55^, Asn312^7.39^ and Asp113^3.32^; SB to Asp113^3.32^; cation–π interaction with Phe193^ECL2^; and a π-stacking interaction with Phe290^6.52^.

We used the DarwinDock complete sampling method to predict the binding sites of the active (Gαs-bound) β_2_AR, as described for other GPCRs (42, 45). DarwinDock samples a large number of poses (∼50,000) in the likely binding regions after replacing the hydrophobic residues with Ala and then chooses the side-chain orientations for these hydrophobic residues separately for each of the best 100 poses (45). Because of the size and the geometry of the atypical C1 ligand, we first docked separately the imidazole and urea subunits, as shown in *SI Appendix*, Fig. S10. We then combined these two portions of the ligand with the cyclohexane–alkyl chain to form the C1-*S* compound, which we minimized using charges from QM (see *Methods* and *SI Appendix*, *Modeling Methods*). The binding sites for the C1-*R* form were predicted in the same fashion as C1-*S*. After incorporating C1 or C5, we reattached the Gs protein and reinserted the ligand–β_2_AR–Gs complex into a lipid membrane and water box at physiological pH and salt. We included the S-palmitoyl-cysteine lipid anchor at the carboxyl terminus of helix 8 (*SI Appendix*, Fig. S11) and the N terminus of Gαs. We then carried out a series of computations (minimization and MD simulations) to predict conformational changes promoted by C1 and C5, providing the energetically favorable receptor conformations expected to be relevant to understanding why C1-*S* is biased, C5-*S* is balanced, and C1-*R* is an antagonist. The final predicted three-dimensional (3D) structure of the activated agonist–receptor–G protein complex (termed Σ_act_) in the membrane is shown in Fig. 7 for C1-*S*. A global view of the complex within the membrane is shown in Fig. 7*A*, while the position of C1-*S* and its interactions as seen from various views are found in Fig. 7 *B*–*E*. These include SB interactions at Asp113^3.32^ and Phe193^ECL2^ and HBs with Ser203^5.42^ and Asn312^7.39^. The intrareceptor HB between Asp113^3.32^ and Tyr316^7.43^ (in common with the epinephrine-bound receptor) was also realized (Fig. 7 *C* and *D*). Each system was equilibrated with MD for 800 ns using GROMACS (see *SI Appendix*, *Modeling Methods*). The last 100 ns for Σ_act_ with C1-*S* and its stereoisomer C1-*R* are shown in *SI Appendix*, Fig. S12 depicting the time-dependent interaction energy with the 11 most important residues and each of the seven transmembrane (TM) regions. We find that the strong SB for C1-*S* to Asp113^3.32^ is more stable than that for C1-*R*. In addition, the HB to Ser203^5.42^ was more stable and had a greater mean magnitude for C1-*S* compared with C1-*R* as was the interaction with Asn312^7.39^. The pharmacophores for the Σ_act_ state of C1-*S* and C1-*R*, and for the unbiased agonist epinephrine, are shown in Fig. 8 *A*–*C*, respectively. The SB to Asp113 is common to C1-*S,* C1-*R*, and epinephrine. The indicated aromatic groups of C1-*S* form a π–π stacking network (Fig. 8*A*) with Phe193^ECL2^, but the analogous interaction is not observed for C1-*R* (Fig. 8*B*). Finally, the HB from C1-*S* to Asn312^7.39^ (Fig. 8*A*) was not observed for C1-*R* (Fig. 8*B*).

### Interactions of C1-*S* and C1-*R* with the Inactive β_2_AR State (Σ_0_).

**Fig. 9.**
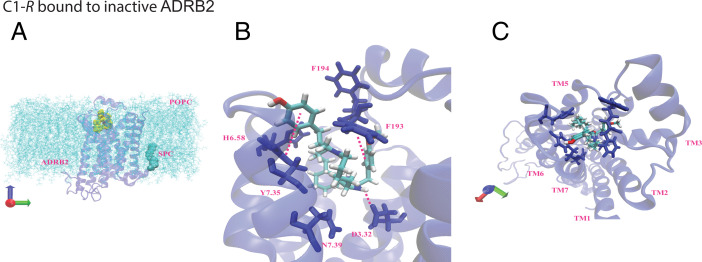
Interactions of C1-*R* with inactive β_2_AR (apo-β_2_AR). (*A*) The minimized inactive β_2_AR (blue)-C1-*S* (yellow) complex in the phosphatidylcholine (POPC) membrane (light blue). (*B* and *C*) The binding pocket of C1-*R* in the inactive β_2_AR includes Asp113^3.32^, His296^6.58^, and aromatic interactions with Phe193^ECL2^, placed in the TM3-4-5-6-7 region. See also the energy plots in *SI Appendix*, Fig. S13 and the pharmacophores in *SI Appendix*, Fig. S14.

Our experiments found evidence that C1-*R* acts as an antagonist. To explore this possibility computationally, we started with the apo-β_2_AR from the high-resolution crystal structure of an engineered inactive human β_2_AR (PDB 2RH1) not complexed to the G protein. We added the S-palmitoyl-cysteine lipid anchor and inserted the apo-β_2_AR into the lipid membrane and solution and then minimized. Then, we inserted the C1*-R* ligand into the apo-β_2_AR and minimized the ligand–Σ_0_ complex, leading to the structures shown in Fig. 9*A* (the complex depicted in the membrane) and two views of the C1-*R* bound to the indicated residues (Fig. 9 *B* and *C*). We carried out MD for 300 ns to equilibrate both structures. Then we carried out 0.5-μs MD simulations of each complex and averaged the interaction energies with the residues and the TMs over the last 300 ns. The interaction energy plots in *SI Appendix*, Fig. S13 show significantly different energy distributions between C1-*S* and C1-*R,* consistent with the 3D structures shown in Figs. 7 and 9. For C1-*R*-Σ_0_ (*SI Appendix*, Fig. S13), we observe interactions with His296^6.58^, Asn293^6.55^, Asn312^7.39^, and strong aromatic interactions with Phe193^ECL2^ and Tyr308^7.35^. Except for Asn312^7.39^, these interactions have greater energies, or are not observed, with C1-*S-*Σ_0_ (*SI Appendix*, Fig. S13). Also, both C1-*S* and C1-*R* retain binding to Asp113^3.32^ in the Σ_0_ state. Taken together, these results indicate that C1-*R* binding to β_2_AR in the Σ_0_ state is energetically more favorable than C1-*S*, which is consistent with the experimental data indicating that C1-*R* fails to activate yet binds to the receptor, thus acting as an antagonist. The pharmacophores for C1-*S* and C1-*R* at Σ_0_ are shown in *SI Appendix*, Fig. S14. The Asp113^3.32^ SB is observed for both ligands. C1-*R* and C1-*S* also both showed stable aromatic bonds to Tyr308^7.35^, but, as indicated in *SI Appendix*, Fig. S13, the energies favor C1-*R* binding. The C1-*R* interaction with Phe193^ECL2^ was not observed with C1-*S* (*SI Appendix*, Fig. S13). The binding site interactions of the inverse agonist carazolol (2RH1) to β_2_AR show several commonalities with those we identified with C1-R to the inactive state, including HBs with Asp113^3.32^, Asn312^7.39^, Tyr316^7.43^, and aromatic interactions with Phe290^6.52^ and Tyr308^7.35^ (46).

### Comparison of Binding Sites of Biased C1-*S* versus Epinephrine and C5-*S* to Σ_act_ β_2_AR.

The differences in the interactions with the activated receptor for the endogenous, unbiased, agonist epinephrine, compared with the biased agonist C1-*S*, are shown in Fig. 8. For Σ_act_, both epinephrine and C1-*S* form SB to Asp113^3.32^; however, epinephrine also interacts with this residue with an HB arising from the β-carbon hydroxyl of the catecholamine (Fig. 8*C*). Both agonists interact with Asn312^7.39^, Ser203^5.42^, and Phe193^ECL2^. However, C1-*S* acts as a proton acceptor at Ser203^5.42^, whereas epinephrine acts as a proton donor at this same residue. In addition, with C1-*S*, a two-aromatic π–π stacking interaction is observed with Phe193^ECL2^, while epinephrine has a cation–π interaction with this residue arising from the terminal amino group of the agonist. Epinephrine also interacts with residues Ser207^5.46^ and TM6 residues Asn293^6.55^ and Phe290^6.52^, which are not present with C1-*S*.

We note that C1-*S* differs from agonist C5-*S* only at the R2 group (*SI Appendix*, Fig. S2), in which C1-*S* has a cyclohexane and C5-*S* has a benzene. Since C1-*S* is biased away from β-arrestin and C5-*S* is not, we modeled C5-*S* interactions with activated receptor to explore the differences between the two compounds that might account for the C1-*S* phenotype (*SI Appendix*, Fig. S15 *A–C*). We find SB interaction with C5-*S* at Asp113^3.32^ and single aromatic π-stacking with Phe193^ECL2^ similar to C1-*S*. However, while C1-*S* shows an HB with Ser203^5.42^, C5-*S* interacts with Ser207^5.46^. In addition, C5-*S* has an HB with Asn293^6.55^ instead of the Asn312^7.39^ residue found for C1-*S*. Indeed, the Asn-293^6.55^ HB and C5-*S* TM6 interaction energies are greater than those found with C1-*S* (compare *SI Appendix*, Figs. S12 and S15*D*). In contrast, the TM7 interaction energies are greater for C1-*S* compared with C5-*S*, primarily as a result of the Asn312 interaction.

The predicted interaction of C1-*S* with Asn312, but not Asn293, while the interaction of C5-*S* is with Asn293 but not Asn312 suggested that mutations of these two residues would have different effects depending on the agonist, which would further confirm the modeling. An Asn293-to-Ala substitution resulted in the loss of high-affinity binding with isoproterenol as determined using ^125^ICYP binding with membranes in the absence of guanosine triphosphate (GTP) (*SI Appendix*, Fig. S16). This result is consistent with catecholamines binding to Asn293 (as well as Asn312; Fig. 8). The mutant Ala293 receptor also lost high-affinity binding compared with wild type (WT) for C5-*S*, in agreement with the modeling indicating C5-*S* binding to Asn293 in the activated state (*SI Appendix*, Fig. S16). It has been previously reported (47, 48) that cAMP responses to isoproterenol with mutations at Asn293 are impaired compared with the WT receptor. Our results with this agonist confirmed these observations, and we also found impaired cAMP signaling with the C5-*S* agonist (*SI Appendix*, Fig. S17). These results are consistent with the modeling and competition studies showing an important C5-*S* interaction with Asn293. The C1-*S* compound did not reveal a high-affinity binding site with the WT receptor (*SI Appendix*, Fig. S16), so this specific phenotype could not be compared with the mutant. Nevertheless, the extent of the competition and the low-affinity K_i_ values were the same with C1-*S* at the Ala293 receptor compared with WT. Importantly, the cAMP responses did not differ between WT and Ala293 for C1-*S*, which is in contrast to C5-*S* (*SI Appendix*, Fig. S17). Collectively, the data suggests dependence on Asn293 for C5-*S*, but not C1-*S*, for β_2_AR activation. Experiments were also considered to explore the TM7 interaction at Asn312. However, the Ala312 mutant receptor failed to bind ^125^ICYP or another antagonist radioligand, ^3^H-dihydroalprenolol, while Western blots verified cell-surface expression of the mutant receptor (*SI Appendix*, Fig. S16*D*). In addition, the cAMP responses with the Ala312 receptor were decreased by >80% and the curves were shifted >2-logs to the right compared with WT (*SI Appendix*, Fig. S17*A*). Taken together, the mutation at Asn312 appears to lead to a distorted binding pocket for antagonist and agonist and was markedly impaired functionally, and thus this receptor was not further studied.

## Discussion

Agonists acting at β_2_AR expressed on ASM cells are the only direct bronchodilators currently available for treating obstructive airways diseases (49). Given that the number of individuals with asthma and COPD exceeds 300 million, the β_2_AR may be the most common GPCR targeted by prescribed agonists worldwide. However, β-agonist treatment has been associated with several outcomes linked to a loss of effectiveness over time. Both short- and long-acting agonists have been associated with tachyphylaxis (16–18), increases in airway responsiveness to constrictive stimuli (50, 51), loss of the bronchoprotective effect (52, 53), and increased morbidity and mortality (54–57). Extensive cellular and biophysical studies of β_2_AR have identified β-arrestin binding as the initial event that results in both rapid attenuation of signaling via Gαs as well as the internalization of receptors leading to long-term down-regulation (58). Thus, a therapeutic that is a Gαs-promoting agonist but biased away from β-arrestin engagement should improve airway response with reduced tachyphylaxis. Biased agonists have been found for several GPCRs including those that are biased toward or away from G protein signaling or β-arrestin actions. For example, the µ-OR agonist TRV130 displays a bias toward G protein (Gαi) coupling and away from β-arrestin. It is equi-effective with morphine in providing analgesia but displays fewer on-target side effects, such as respiratory depression, which has been attributed to the biasing (59). Indeed, we find that the binding sites for TRV130 on µ-OR differ from those of morphine in several key regions (11). We also note that at the α_2C_AR, the agonists UK14304 and B-HT920 share little structural similarity, yet both are biased toward Gi coupling and away from Gs coupling (7). Such results indicate that quantitatively similar biasing for a given receptor by agonists can be achieved from structurally distinct compounds. Biasing toward β-arrestin and away from G protein coupling can also be attained, which is considered therapeutically favorable for certain receptors and diseases (60). For example, TRV027 (also known as TRV120027) has been shown to be a β-arrestin biased type 1 angiotensin II receptor agonist (60) lacking significant signaling to Gαq. The biased activation of this receptor increased cardiomyocyte contractility in vitro and, in vivo, lowered blood pressure but displayed preserved cardiac output (unlike unbiased agonists) (60–62). Other β-arrestin biased agonists at this receptor, such as SII, have been shown to improve cardiac contractility (63). These effects have been linked to β-arrestin–mediated activation of ERK1/2, Src, Akt, PI3 kinase, and endothelial nitric oxide synthase (64, 65). Given that chronic Gαq activation in the heart exacerbates heart failure (66), such selective agonists may be useful in certain cases for treating hypertension or cardiomyopathies.

In the current study, we sought a β_2_AR agonist biased toward Gαs coupling and away from β-arrestin engagement, with the goal of improving treatment for obstructive lung disease while establishing the structural basis for pathway selectivity for this receptor. We screened a 40-million-compound library ranked by scaffold structure that was impartial to known ligand structures for β_2_AR agonists, with subsequent assessment of agonist propensity to evoke β-arrestin engagement by individual compounds. One compound (C1-*S*) was particularly notable for the apparent absence of β-arrestin engagement while displaying Gαs coupling to cAMP production as well as ASM relaxation. Since there was no detectable β-arrestin signal with C1-*S*, the bias factor β cannot be calculated, but clearly, the presence of Gs coupling by C1-*S*, and the absence of a β-arrestin signal within the limitations of the multiple assays, places this agonist as strongly biased away from the β-arrestin event. The functional evidence to corroborate this biasing phenotype would be minimal short-term, agonist-promoted desensitization. This was examined in our studies in two different ways. First, we measured any loss of the β_2_AR cAMP response after pretreatment with C1-*S* or the positive control agonist albuterol. While the balanced agonist albuterol evoked desensitization, C1-*S* did not (*SI Appendix*, Fig. S6). In a second confirmation of the C1-*S* biasing phenotype, we studied a biologically relevant physiologic response (relaxation) of HASM cells, whose β_2_AR are therapeutic targets for β-agonists. We verified that C1-*S* is an agonist in this system and then found that pretreatment with C1-*S* for 30 min (or 4 h) evoked no desensitization of the relaxation response while albuterol promoted a loss of β_2_AR function (Fig. 6). In a third confirmation, we assessed another functional outcome of β_2_AR-mediated binding of β-arrestin, cAMP-independent activation of ERK1/2. A traditional full and partial agonist promoted ERK1/2 activation, while C1-*S* did not (Fig. 5*H*). Taken together, results from three different types of experiments measuring β-arrestin interaction with β_2_AR (Fig. 5 *A–H*) and three different types of experiments measuring the functional consequences of β-arrestin binding (Figs. 5*H* and 6 and *SI Appendix*, Fig. S6) were congruent. Thus, we conclude with a strong degree of confidence that C1-*S* is biased away from β-arrestin yet maintains Gs coupling sufficient to relax ASM cells. To our knowledge, this direction of biasing for a β_2_AR agonist has not been previously reported. Salmeterol and formoterol, long-acting β-agonists with extended R-group substitutions, have been studied and found to be biased toward β-arrestin (8, 32), which, in the current context, would not be considered clinically favorable. Differences in the assays may explain some discrepancies with regard to salmeterol (8, 32, 47). Carvedilol, classified as a β_1_/β_2_-antagonist with inverse agonist activity at β_2_AR, weakly recruits β-arrestin but does not stimulate cAMP (34). By definition, this could be termed “biasing,” but regardless, it is toward β-arrestin.

Our MD simulations revealed an energetically favorable conformation of C1-*S* with strong interactions with Asp113^3.32^ and Ser203^5.42^, both of which are observed with agonists that activate Gs coupling to the β_2_AR (44, 67, 68). C1-*S* displays an HB with Ser203^5.42^ but not with Ser207^5.46^, which also interacts with epinephrine (44, 67, 68). This absence of the Ser207^5.46^ HB may alter the inward bulge of TM5 that occurs with epinephrine binding, which is associated with the outward movement of TM6 (44, 67). Epinephrine also binds to Phe290^6.52^ in TM6, but C1-*S* does not. Furthermore, the active epinephrine-bound receptor displays HBs between Asn293^6.55^, Ser203^5.42^, Ser207^5.46^, and the hydroxyl groups of catecholamines, as well as an intrareceptor HB between Ser204^5.43^ and the Asn293^6.55^. These HBs have been proposed to be part of a polar network for this balanced agonist (47). In contrast, the C1-*S*–bound receptor only exhibits the Ser203^5.42^ HB. Interestingly, mutation of Ser204^5.43^ to Thr or Ala, which disrupts this network by eliminating the intrareceptor HB, markedly decreases epinephrine-promoted β-arrestin binding to β_2_AR, with a smaller effect on Gs coupling (47). Thus, the C1-*S* signaling phenotype may also be influenced by an atypical polar network between TM5 and TM6, since it lacks the HB with Asn293^6.55^ as well as the intrareceptor HB. In our studies with R-group substitutions on the selected scaffold, we found a compound (C5-*S*) similar to C1-*S* that did not display bias. The two compounds differ only by the R2 group (benzene or cyclohexane, respectively). Comparing the interactions of C5-*S* agonist with activated β_2_AR with those of C1-*S*, we see a potential reorientation of the C5-*S* agonist due to benzene being more exposed to solvent. This appears to occur because the imidazole for C5-*S* makes a strong simultaneous interaction with Asp113^3.32^ in TM3 and Asn293^6.55^ in TM6, which pulls TM3 and TM6 closer in the upper part of the receptor. C1-*S* acted in a similar fashion with the imidazole, but it binds to Asp113^3.32^ and Asn312^7.39^, linking TM3 with TM7 instead of TM6. The net result is a shift of the C1-*S* position away from the TM3-5-6 pocket that forms with C5-*S*, which may contribute to the differences in β-arrestin activation promoted by the two compounds.

In summary, by screening a nontargeted scaffold library of 40 million compounds, we identified the C1-*S* β_2_AR agonist that is biased away from β-arrestin engagement and yet still actively couples to Gs, stimulating cAMP and relaxing ASM. We anticipated that if an agonist could be found that selectively splits the β-arrestin and Gs signals to avoid the actions of β-arrestin, it would act without the tachyphylaxis observed with typical β-agonists. Indeed, studies of C1-*S* with cAMP accumulation or HASM cell relaxation show a lack of tachyphylaxis, representing a favorable pharmacologic effect for treating obstructive lung diseases. The basis of this biasing may be multifactorial, including the loss and gain of binding sites that may affect a hydrophobic network and a positioning of the agonist in the binding pocket that is shifted away from TM6.

## Methods

### Cell Culture, Transfections, and cAMP Assays.

CHW cells were transfected to stably express the human β_2_AR under G418 selection and maintained in monolayers as previously described (69). Transient transfections of HEK-293T cells with constructs to express human β-arrestin2–myc, human β-arrestin2–GFP, human β_2_AR, and β_2_AR-GFP were performed as previously described (31, 70, 71) using Lipofectamine 2000 (Invitrogen) and studied 48 h later. CHO cells stably expressing a modified β_2_AR and a modified β-arrestin2 for the ECA were purchased from DiscoverX. In some experiments, these cells were transiently transfected with 5 μg GRK2 cDNA as described (72). Primary HASM cells were derived from donor lungs obtained from the National Disease Exchange Registry, maintained as monolayers as previously reported (37), and utilized between passages 3 and 6 for the physiological studies. Other experiments used the D9 telomerase reverse transcriptase immortalized HASM cell line as described (70).

### Chemical Libraries.

The SR and PS libraries consisted of mixtures of compounds in wells and were designed as previously reported in detail (26, 29, 30), with the SR library having ∼40 million compounds and the PS library having fewer compounds (and fewer compounds per sample well), based on the chosen candidate SR library sample. Composition of the SR library has been previously published (26). The PS library was custom designed by selecting the multiple moieties at each R1, R2, and R3 position and then synthesizing the combinations of all of these for the next round of cAMP stimulation tests. We ordered the samples by each of the three positions from the cAMP responses and the results assessed by computational methods for trends in structure activity relationships (73).

### Radioligand Binding, cAMP, and ERK1/2 Activation Assays.

For radioligand competition studies, β_2_AR-expressing cell membranes were incubated with ^125^ICYP (40 pM) in 75 mM Tris (pH 7.4), 12 mM MgCl_2_, and 2 mM ethylenediaminetetraacetic acid (EDTA) buffer with or without 100 μM GTP as indicated and the varying concentrations of compounds for 1.5 h at 25 °C as described (69). Bound ^125^ICYP was separated from free radioligand by vacuum filtration over glass fiber filters with 5 mM Tris (pH 7.4) and 2 mM EDTA buffer at 4 °C using a cell harvester (Brandell). For cAMP experiments, β_2_AR-transfected and nontransfected cells were plated onto 96-well plates at 20,000 cells per well in Dulbecco’s modified Eagle’s media without serum and the next day treated with the phosphodiesterase inhibitor 3-isobutyl-1-methylxanthine (100 μM for 30 min). cAMP production from the cells was initiated by exposure to various agents for 10 min at 37 °C, and the reaction was stopped by cell lysis. cAMP was measured by a competitive immunoassay (Molecular Devices) as described (15). ERK1/2 activation was determined in HASM cells by first treating cells with the PKA inhibitor H89 (10 μM) and then exposing the cells for 5 min at 37 °C with vehicle, 10 μM isoproterenol, 300 μM albuterol, or 100 μM C1-*S*, and the proteins were separated by 12% sodium dodecyl sulfate–gel electrophoresis and transferred as described (74).

### β_2_AR:β-arrestin Interactions.

The PLA (Duolink, Sigma) was performed as recently described (31). Briefly, HEK-293T cells were transfected on coverslips with β_2_AR-GFP and β-arrestin2–myc. Primary antibodies to each tag are incubated with transfected cells, and then a pair of oligo-coupled secondary antibodies are added to the dish. Hybridizing oligos connect the two antibody-coupled oligos when they are in close proximity. A signal is generated after a ligase forms circular DNA, which is then amplified by rolling-circle PCR. Fluorescent-labeled oligos hybridize to this product. Cells were exposed to vehicle or the indicated concentrations of agonists for 10 min at 37 °C and fixed with 4% paraformaldehyde. Cells were imaged by fluorescence confocal microscopy. The ECA (PathHunter, DiscoverX) was performed as another approach to detect receptor β-arrestin interaction (32). Briefly, attached CHO cells, stably transfected to express a β-arrestin2 fused to a β-galactosidase that lacks a peptide fragment, and a β_2_AR that is tagged at its carboxyl terminus with the complementary β-galactosidase fragment, were studied in 96-well plates. Agonists were incubated with the cells for 30 min at 37 °C. Upon agonist-promoted binding of β-arrestin to receptor, the β-galactosidase is reconstituted to yield active enzyme, which is detected by luminescence on a FlexStation3 plate reader. The β-arrestin–GFP based recruitment assays were performed as previously described (15). 

### BRET2 Gαs Activation Assays.

Gαs activation by β_2_AR was quantitated by determining the dissociation of the Gs heterotrimer using BRET2. HEK-293T cells were transfected using Lipofectamine 2000 with cDNA constructs encoding β_2_AR, the short form of Gαs fused to RLuc8, Gβ, and Gγ fused to GFP2 (Addgene) at a ratio of 1:1:1:1, as described by Olsen et al. (35). The next day, they were lifted and plated on 96-well plates, and 48 h after transfection, the RLuc8 substrate was added and cells were treated with multiple doses of agonist in quadruplicate for 5 min at room temperature. The Rluc8 signal was acquired at 395 nm and the GFP2 signal at 510 nm on a FlexStaion3. BRET2 was calculated as the ratio of the GFP2 to RLuc8 signals. 

### MTC.

We used MTC to measure dynamic changes in the cytoskeletal stiffness as a surrogate for agonist-induced single-cell relaxation, as we have validated previously (32, 33, 37, 38). Ferrimagnetic microbeads (4.5 μm in diameter) coated with synthetic peptides containing Arg-Gly-Asp (RGD) were ligated to cell-surface integrin receptors that form focal adhesions and are tightly linked to the underlying cytoskeleton network (Fig. 6*A*). Beads were magnetized horizontally to cell plating and then twisted in a vertically aligned magnetic field that varied sinusoidally in time. Forced bead motions were detected optically with a spatial resolution of ∼5 nm, and their changes monitored, in real time, in response to β-agonist (HASM cell relaxation). The greatest relaxation change from baseline observed at any point over the time course was utilized to quantify the maximal HASM response to isoproterenol.

### Ligand–Receptor Structural Analysis and Molecular Docking.

We began by incorporating the data from the β_2_AR crystal structure (PDB 3SN6). The DarwinDock complete sampling method was utilized to predict the binding sites and energetics of C1-*S*, C1-*R*, and C5-*S* bound to β_2_AR as we have described previously for other GPCRs (41–43, 45) (*SI Appendix*, *SI Expanded Methods*). To define the sites and energetics for the activated receptor (Σ_act_), the ligand–receptor–Gs complex was modeled, while for the inactive state (Σ_0_), the ligand–receptor complex in the absence of G protein was modeled. MD simulations were carried out using GROMACS (Uppsala University) as described (41), with optimization as indicated in *SI Appendix*, *SI Expanded Methods*.

### Data Analysis and Statistical Comparisons.

cAMP values from the screens were compared by ANOVA followed by post hoc *t* tests with Tukey’s correction for multiple comparisons. The basal (Rmin), maximal (Rmax), the concentration resulting in half maximal response (EC_50_), the Hill coefficient, and the R^2^ of the fit for each concentration-response curve was obtained by iterative four-parameter least-squares logistic regression fitting to a sigmoid curve using Prism (GraphPad). The Emax (Rmax-Rmin) and EC_50_ values were compared by *t* tests using the same software. For the PLA, ECA, and Gs-activation studies, data were fit to a three-parameter logistic function with Hill coefficient set at 1.0. The bias factor was calculated from the Gαs activation and ECA data using the logistic equiactive method (method 3 of ref. 32, see equation in *SI Appendix*, *SI Expanded Methods*). Other data as indicated were compared by *t* tests with Tukey’s correction for multiple comparisons. *P* values of <0.05 were considered statistically significant. Data from multiple experiments are shown as mean ± SE unless otherwise indicated.

## Supplementary Material

Supplementary File

## Data Availability

All study data are included in the article and/or *SI Appendix*.
