# Supplementary material for: Identification and characterization of an atypical Gαs-biased β2AR agonist that fails to evoke airway smooth muscle cell tachyphylaxis
Source: Proc Natl Acad Sci U S A. 2021 Dec 2;118(49):e2026668118. doi: 10.1073/pnas.2026668118 (PMC8670521; doi:10.1073/pnas.2026668118)
Supplement: Supplementary File [file pnas.2026668118.sapp.pdf]

PNAS
www.pnas.org

Supplementary Information for:

Identification and characterization of an atypical $G\alpha s$-biased β_2AR agonist that fails to evoke airway smooth muscle cell tachyphylaxis

Donghwa Kim[a,1], Alina Tokmakova[b,1,3], Lauren K. Lujan[a], Hannah R. Strzelinski[a], Nicholas Kim[c], Maliheh Najari Beidokhti[a], Marc A. Giulianotti[d], Amirhossein Mafi[b], Jung-A A. Woo[e], Steven S. An[c], William A. Goddard III[b,2], Stephen B. Liggett[a,e,2]

[a] Department of Medicine, University of South Florida Morsani College of Medicine, Tampa FL, 33612

[b] Materials and Process Simulation Center, California Institute of Technology, Pasadena CA, 91125, current address: Program in Biophysics, University of California San Francisco, 94102

[c] Department of Pharmacology, Rutgers-Robert Wood Johnson Medical School and the Rutgers Institute for Translational Medicine and Science, New Brunswick, NJ 08901

[d] Center for Translational Science, Florida International University, Port St. Lucie, FL, 34987

[e] Department of Molecular Pharmacology and Physiology, University of South Florida Morsani College of Medicine, Tampa, FL, 33612

[1] Contributed equally to this work

[2] To whom correspondence may be addressed: wag@caltech.edu; sliggett@usf.edu

ORCID WAG:0000-0003-0097-5716; SBL:0000-0002-0128-3669

[3] Present address: Program in Biophysics, University of California, San Francisco, CA 94102

Corresponding authors: S. Liggett (sliggett@usf.edu) or W. Goddard (wag@caltech.edu)

This PDF file includes:

Figures S1 to S17
Expanded Methods
SI References

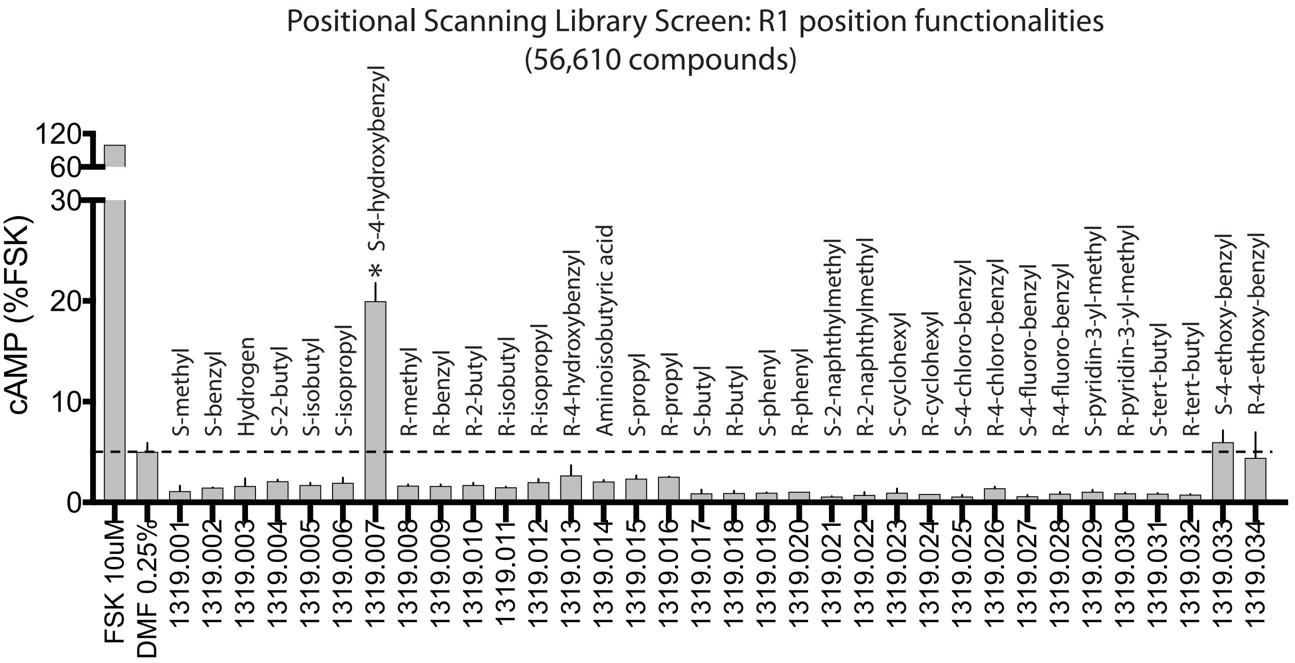

Fig. S1. Results from cAMP screening of CHW-β_2 cells with a 56,610 compound positional scanning library with all potential R1 moieties in the library. Each well contained 1,665 compounds. Only well 1319.007 showed a significant increase over baseline (the DMF carrier). Shown are results from 3 experiments performed. *, P<0.01 vs DMF.

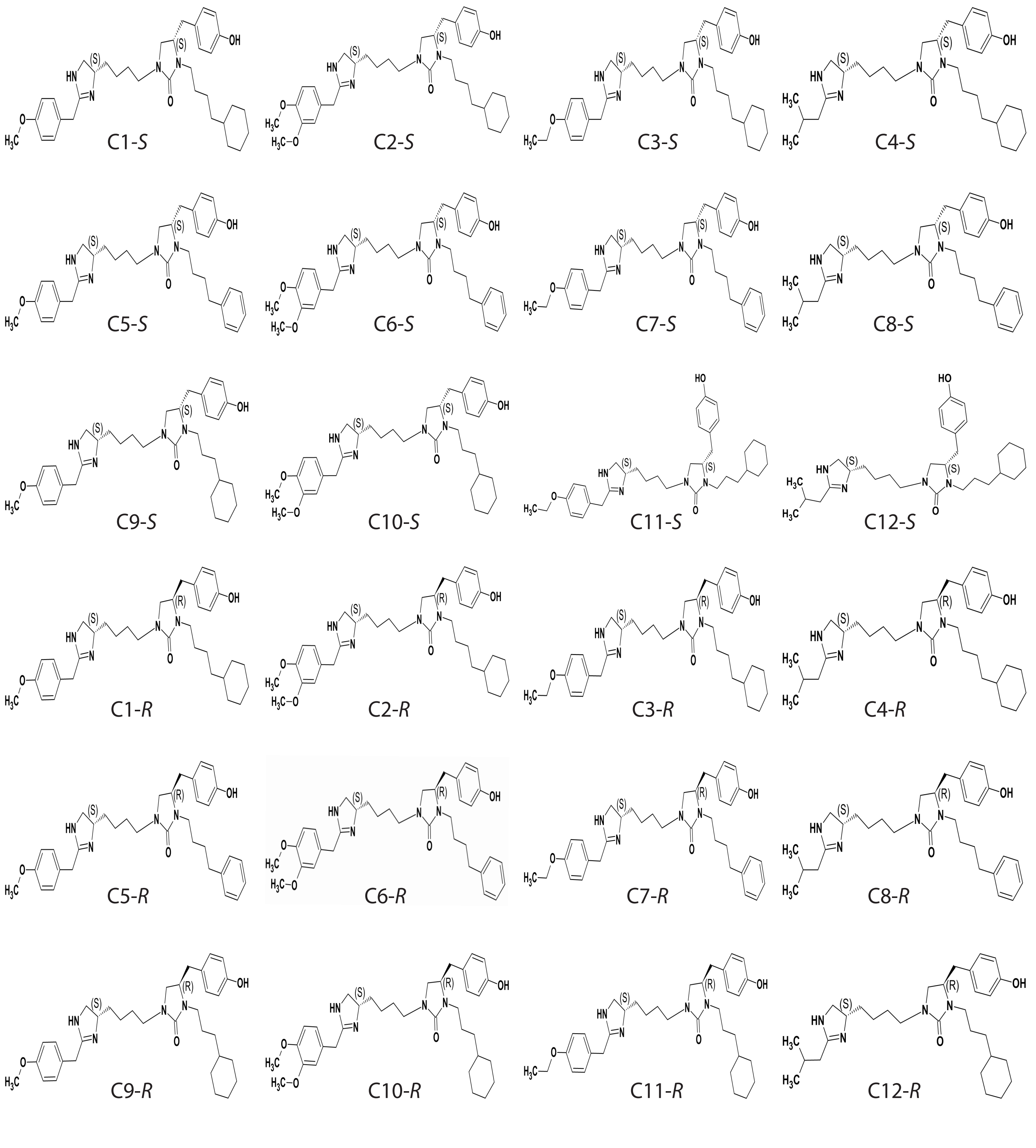

Fig. S2. Structures of C1–C12-*S* and C1–C12-*R*. The backbone structure with the three R groups is shown in Fig. 2A.

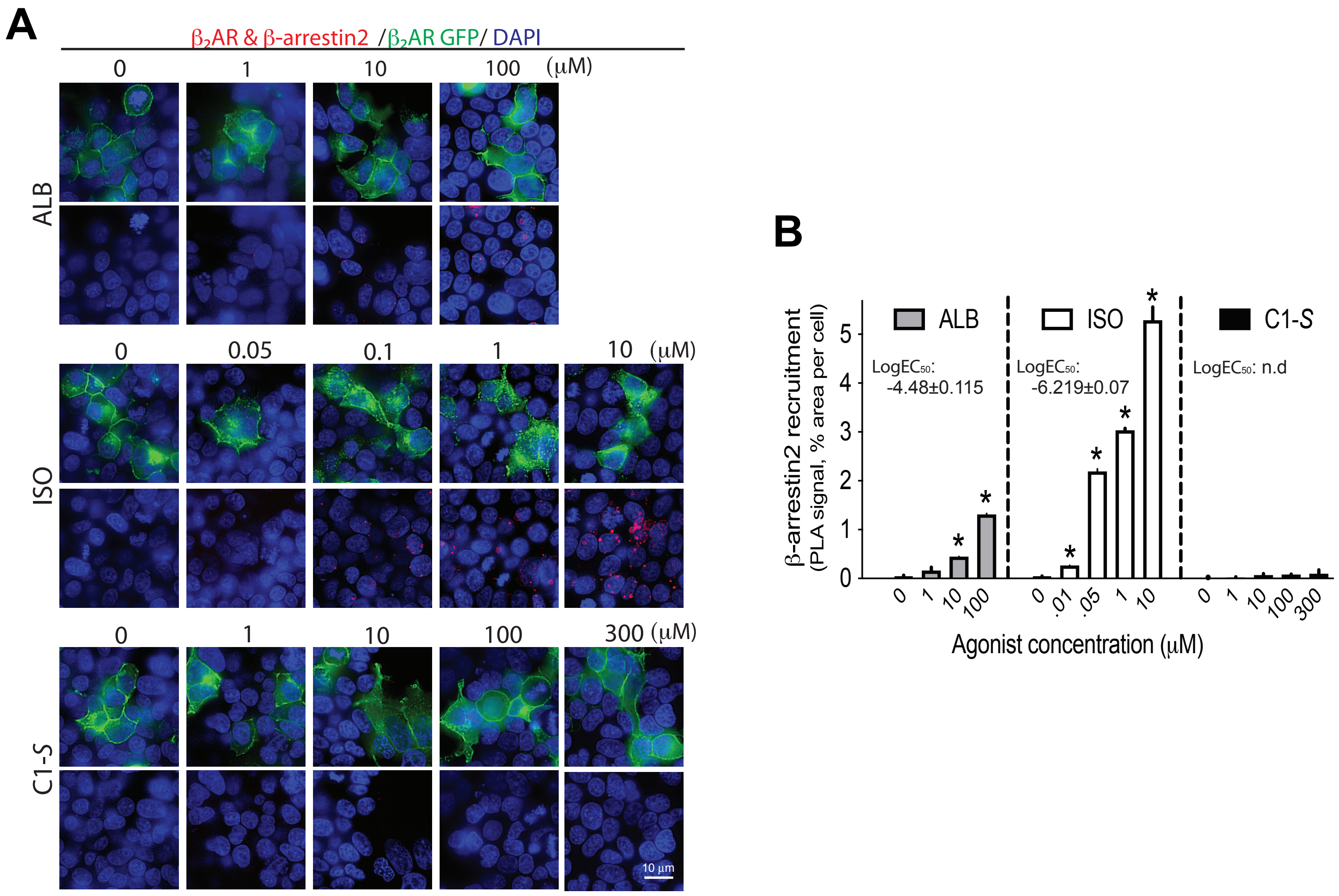

Fig. S3. C1-*S* fails to promote β_2AR binding to β-arrestin as determined by a PLA. Transfected cells (see Methods) were exposed to the agonists albuterol (ALB), isoproterenol (ISO), and C1-*S* at the indicated concentrations for 10 min. (A) Representative experiment, of 4 performed, visualized by confocal microscopy. The red signal indicates β-arrestin binding to the receptor. (B) Results from 4 experiments with the mean ± SE of the maximal response and the EC_{50} indicated for each agonist. There was no β-arrestin response from C1-*S*, so the EC_{50} was not determined (n.d.). * P<0.01 vs vehicle control.

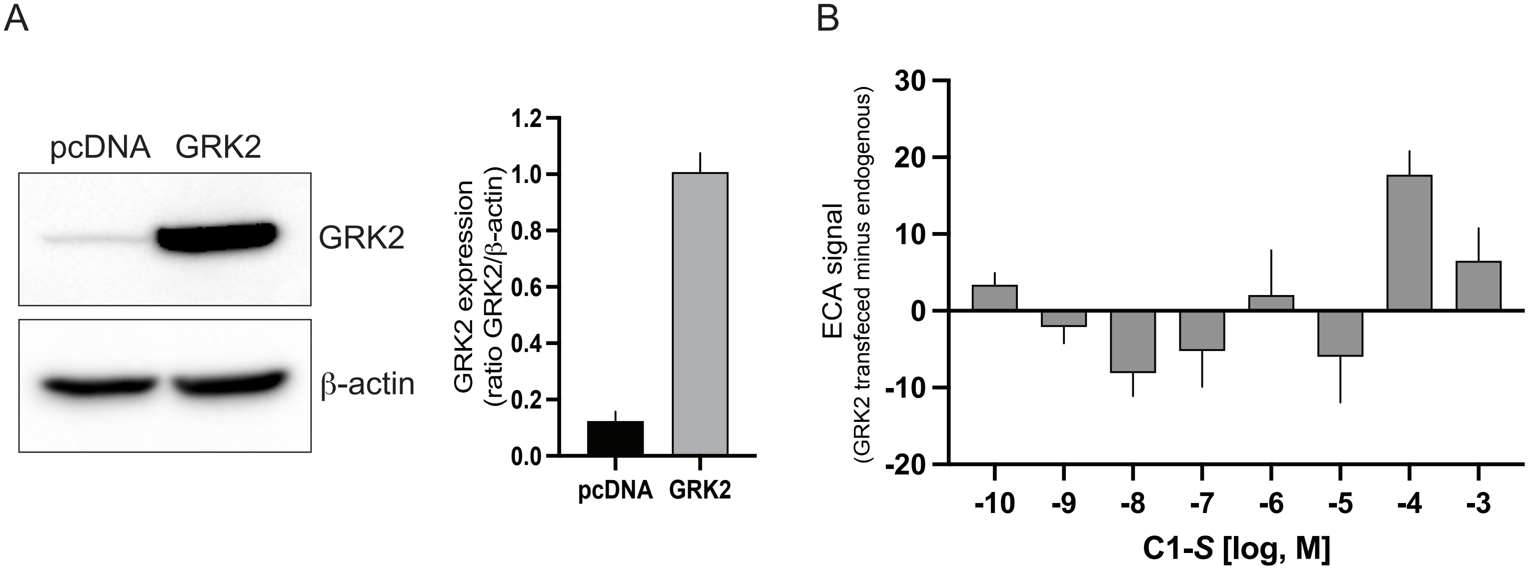

Fig. S4. GRK2 overexpression does not increase C1-*S* promoted β-arrestin binding to β2AR as determined by the ECA. (A) Stably transfected CHO cells (PathHunter, see Methods) expressing modified β_2AR and β-arrestin were transiently transfected with GRK2 to achieve > 8 fold overexpression compared to endogenous GRK2. (B) Parallel ECAs were performed without or with GRK2 overexpression using the indicated concentrations of C1-*S* . Similar to what was found in experiments shown in Fig. 5F, the responses were not consistently above vehicle control and a dose-response curve could not be fit. The differences in the ECA signal between cells overexpressing and endogenously expressing GRK2 were determined at each concentration (set as the y-axis). These differences fluctuated and included both positive and negative values, and ANOVA failed to show significance. Results are from 4-6 experiments.

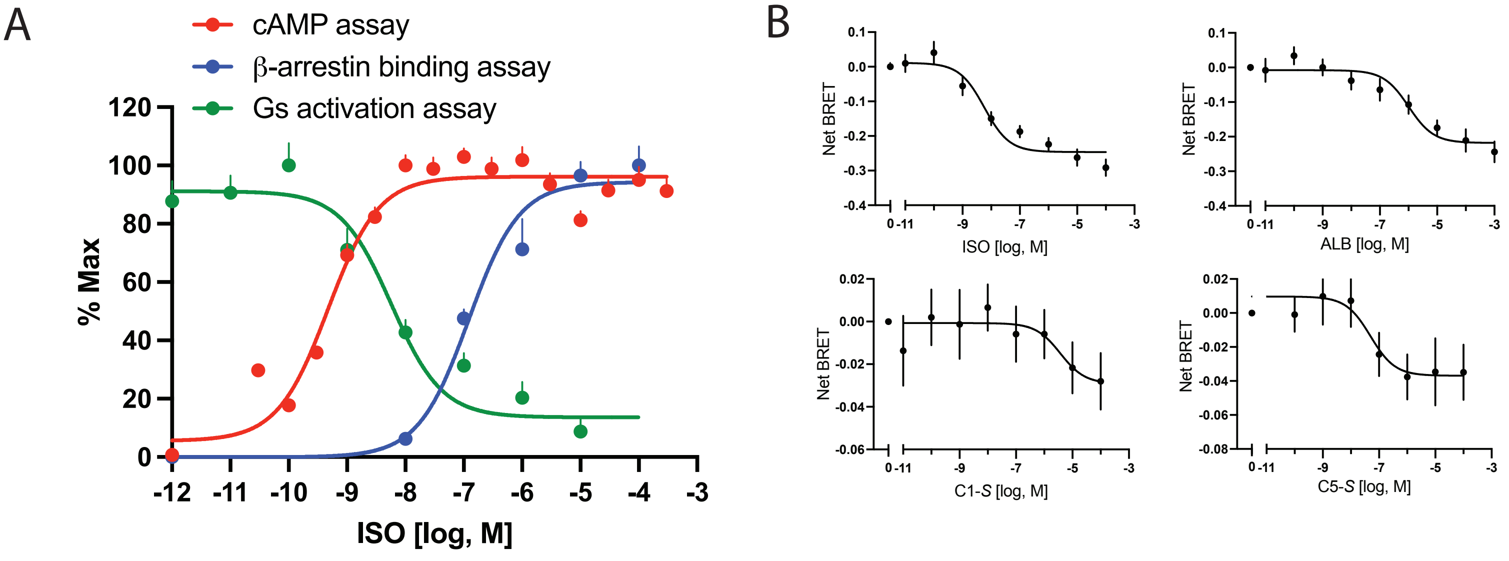

C

β-arrestin binding (ECA)				
	$LogEC_{50}$	SEM $LogEC_{50}$	Emax	SEM Emax
ISO (n=3)	-6.962	0.07	1288.1	43.01
ALB (n=8)	-5.745	0.125	66.14	3.91
C1-*S* (n=4)	UND	UND	UND	UND
C5-*S* (n=4)	-6.030	0.454	18.9	3.81
**Gs coupling (BRET2)**				
	$LogEC_{50}$	SEM $LogEC_{50}$	Emax	SEM Emax
ISO (n=6)	-8.241	0.153	0.258	0.015
ALB (n=6)	-5.997	0.174	0.210	0.015
C1-*S* (n=4)	-6.219	0.485	0.049	0.011
C5-*S* (n=4)	-7.255	0.792	0.041	0.013

Fig. S5. Additional properties of C1-*S* and C5-*S* acting at the β_2AR. (A) Data from cAMP accumulation, Gs-activation, and β-arrestin binding experiments with ISO are plotted together. The BRET2-based Gs-activation responses represent the dissociation of the Gs heterotrimer upon coupling of the α-subunit to the receptor, and the values decrease with higher concentrations of agonist. The data indicates for this full, unbiased, reference agonist, that the Gs-activation assay more closely mirrors the β-arrestin binding assay in terms of EC_{50}. Results are from 4 experiments. (B) Gs activation (BRET2 assay) responses to ISO, ALB, C1-*S* and C5-*S*. Results are from 4-6 experiments. (C) Summary of the β-arrestin binding and BRET2 results. Emax is the net change in a signal (Rmin-Rmax).

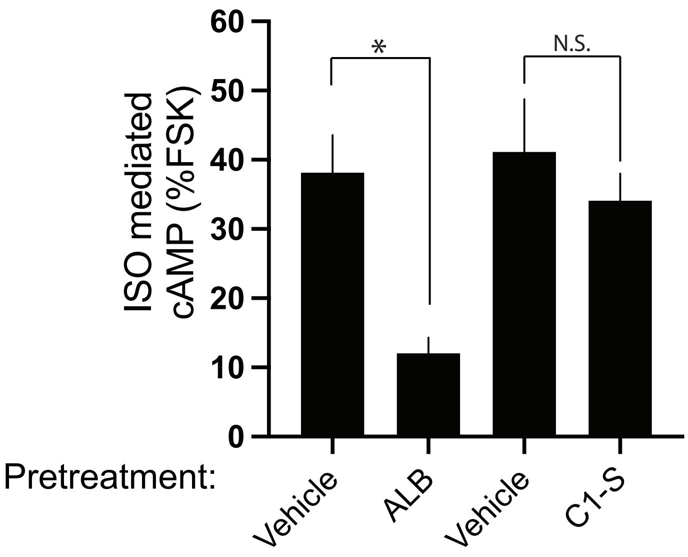

Fig. S6. C1-*S* does not promote short-term agonist mediated desensitization of β_2 AR. Attached CHO-β_2 cells were exposed to vehicle, 10 µM albuterol (ALB) or 150 µM C1-*S* for 10 min, washed, and the cAMP response to 10 µM isoproterenol (ISO) determined after a 10 min incubation. As shown, ALB pretreatment resulted in ~68% desensitization of the subsequent ISO response. In contrast, C1-*S* pretreatment caused no desensitization. *, P<0.01 vs vehicle, N=4 experiments

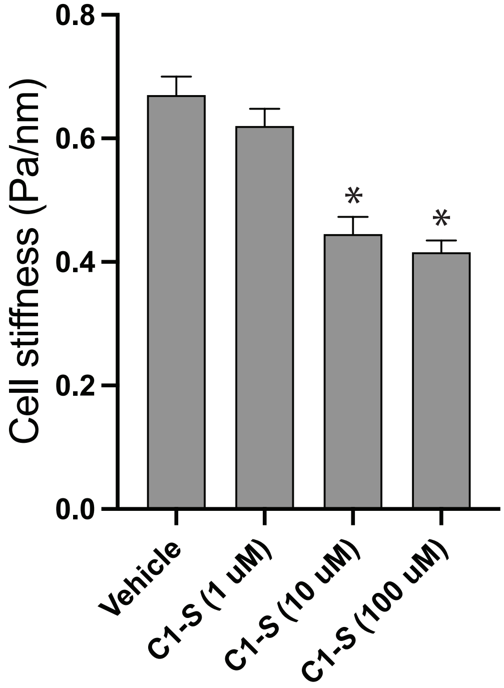

Fig. S7. C1-*S* relaxes human airway smooth muscle cells. MTC (see Methods and Fig. 6A) was utilized to measure cell stiffness (shown as raw values), where a decrease in stiffness represents relaxation. The maximal response at the three indicated concentrations are shown. *, $P<0.01$ vs vehicle, N=4 experiments.

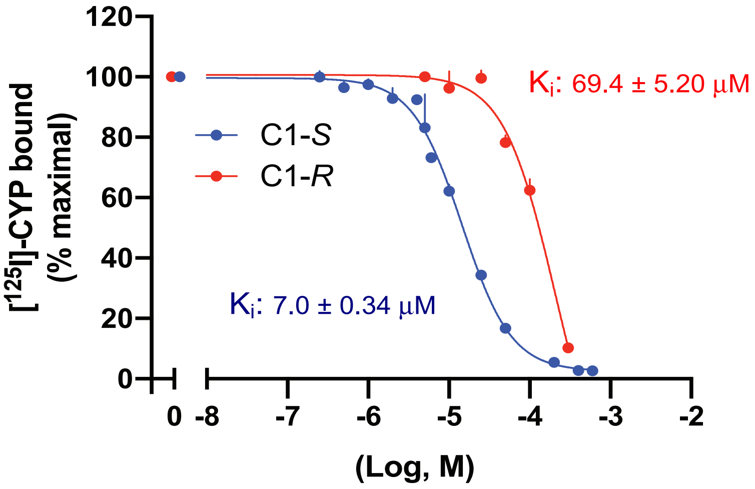

Fig. S8. Competition between C1-*S* or C1-*R* with the βAR radioligand ^{125}I-CYP for β_2AR expressed on cell membranes. The stable CHW-β_2 cell line was used to prepare cell membranes and competition studies were performed with 40 pM ^{125}I-CYP in the presence of 100 µM GTP. Results are from 3 experiments.

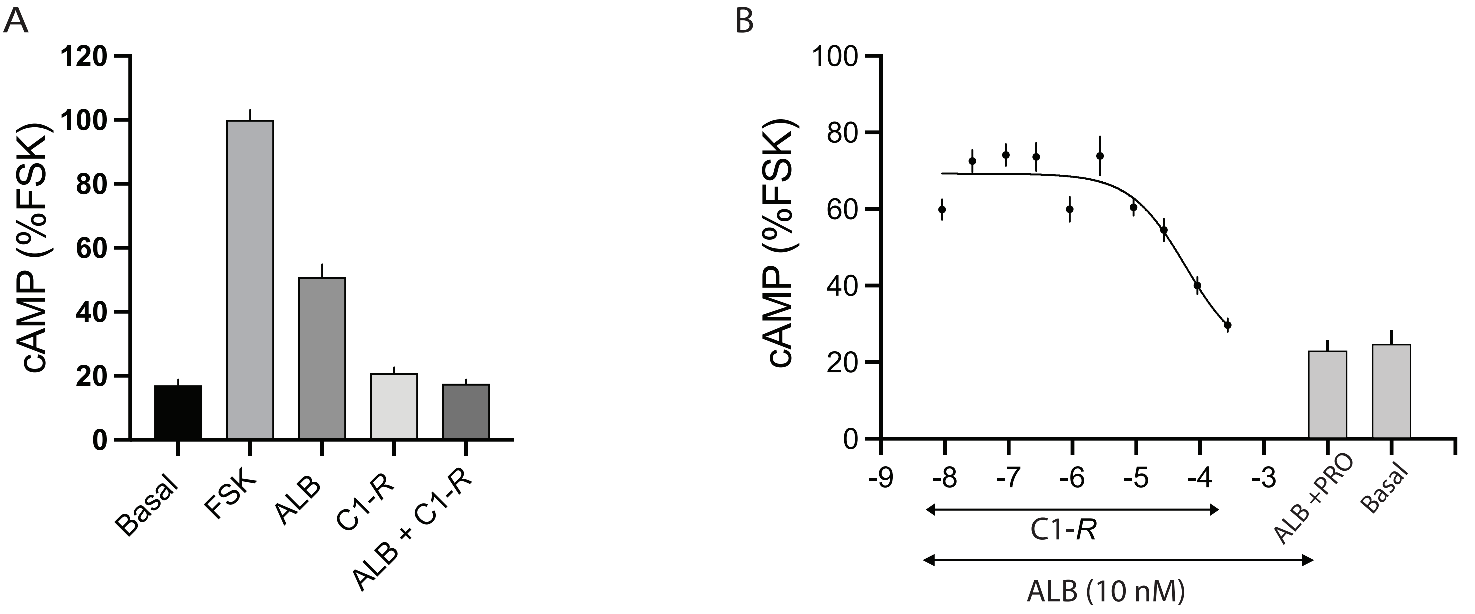

Fig. S9. C1-*R* acts as a functional antagonist at β_2AR. (A) Intact CHW-β_2 were exposed for 10 min to vehicle (basal), 5 µM forskolin (FSK), 10 nM albuterol (ALB), or 10 nM albuterol + 300 µM C1-*R* and cAMP levels determined. There was no difference between Basal and ALB + C1-*R* cAMP levels. (B) Dose-response of C1-*R* for antagonizing ALB-promoted cAMP. Propranolol (PRO, 1.0 µM) served as a known antagonist. N=3-4 experiments.

C1

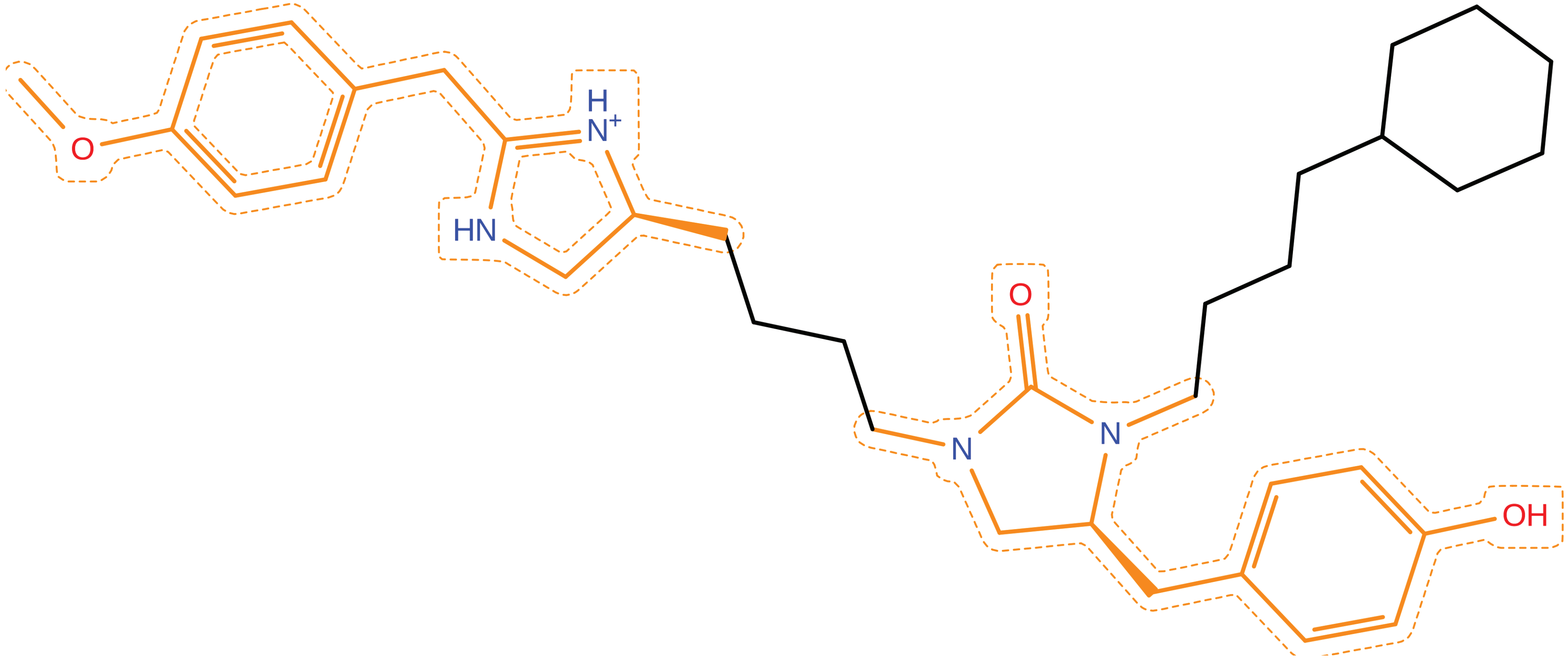

Fig. S10. The C1 compound was divided into two main parts (orange) which were docked to the receptor separately. The whole ligand was reconstructed by connecting all parts (black and orange) together while retaining the strong interactions with the protein.

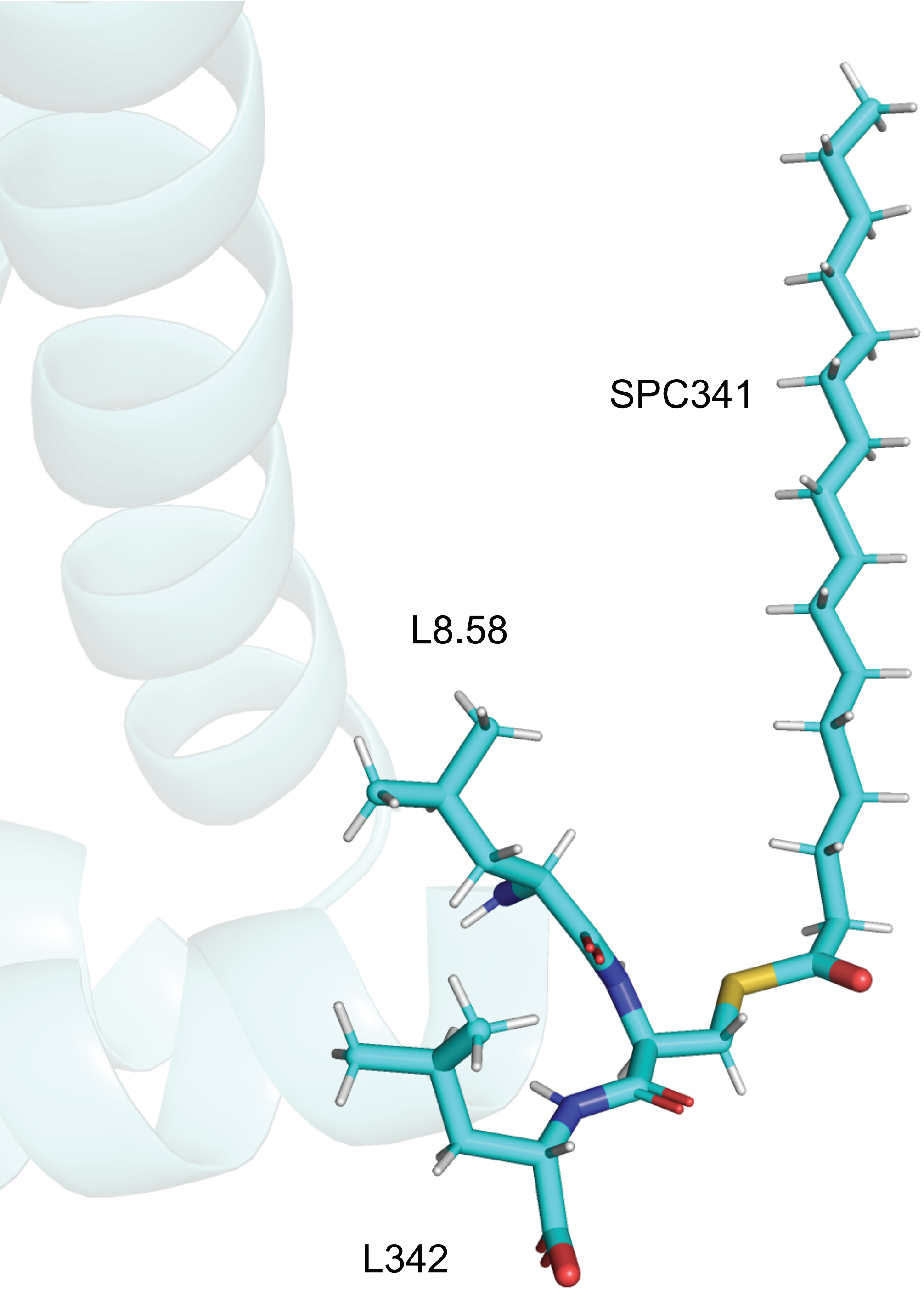

Fig. S11. The lipid modifications of the activated receptor were made by modeling palmitoyl-cysteine 341 and palmitoyl-cysteine 3 in the Gαs subunit (not shown).

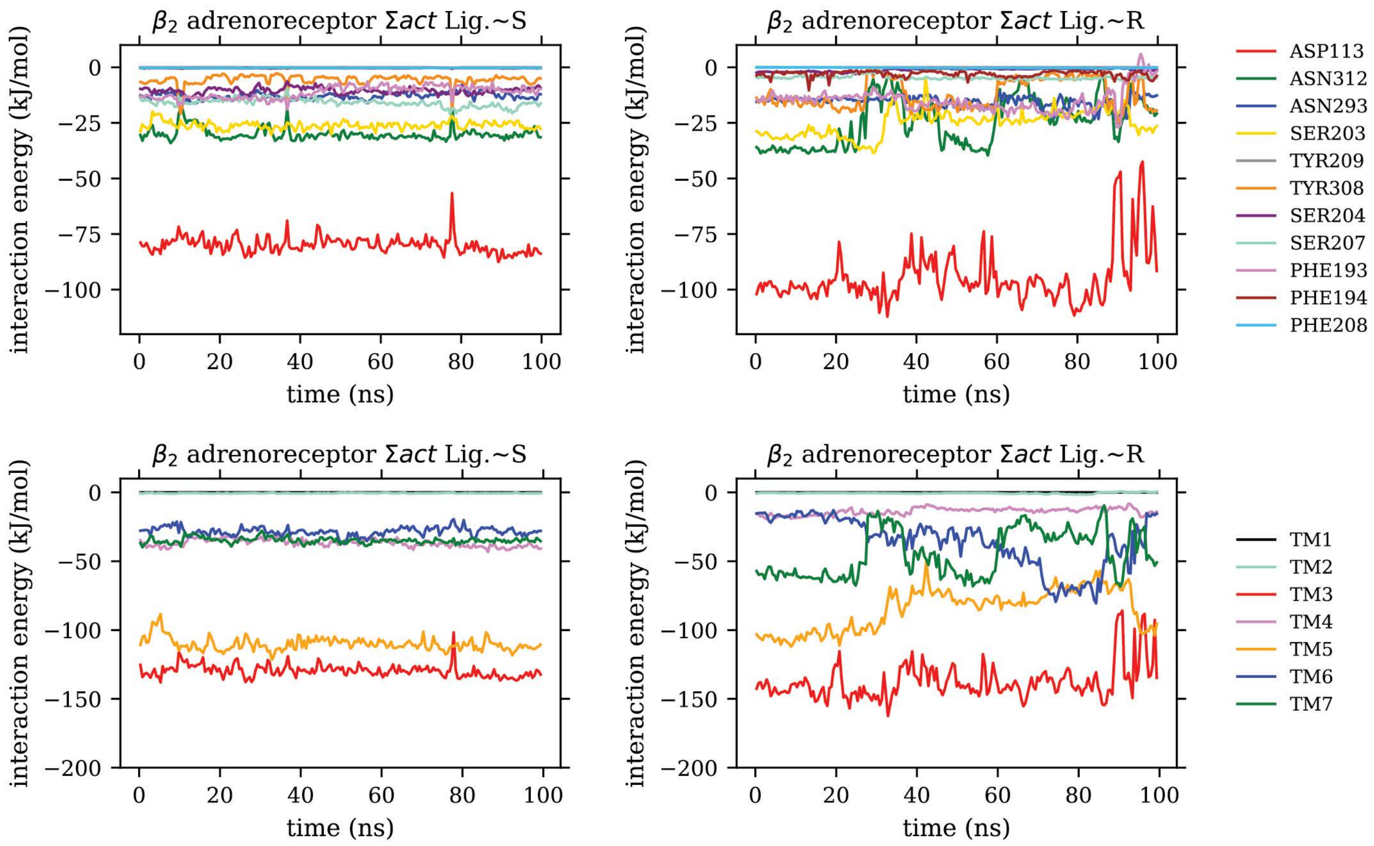

Fig. S12. Time evolution for interaction energies of various β_2AR residues in the active state with the C1-*S* and C1-*R* compounds. The two upper plots show the interaction energies between the ligand (left –C1-*S*, right –C1-*R*) and 11 receptor residues within the radius of 5 Å from the ligand in the active state. The two lower plots show the interaction energies between the ligand (left –C1-*S*, right –C1-*R*) and the 7 TM regions of β_2AR in the active state. Collectively these results indicates binding of C1-*S* that is more stable (Asp113$^{3.32}$, red) or of greater average magnitude (Ser203$^{5.42}$, yellow) and (Asn312$^{7.39}$, green) compared to C1-*R*.

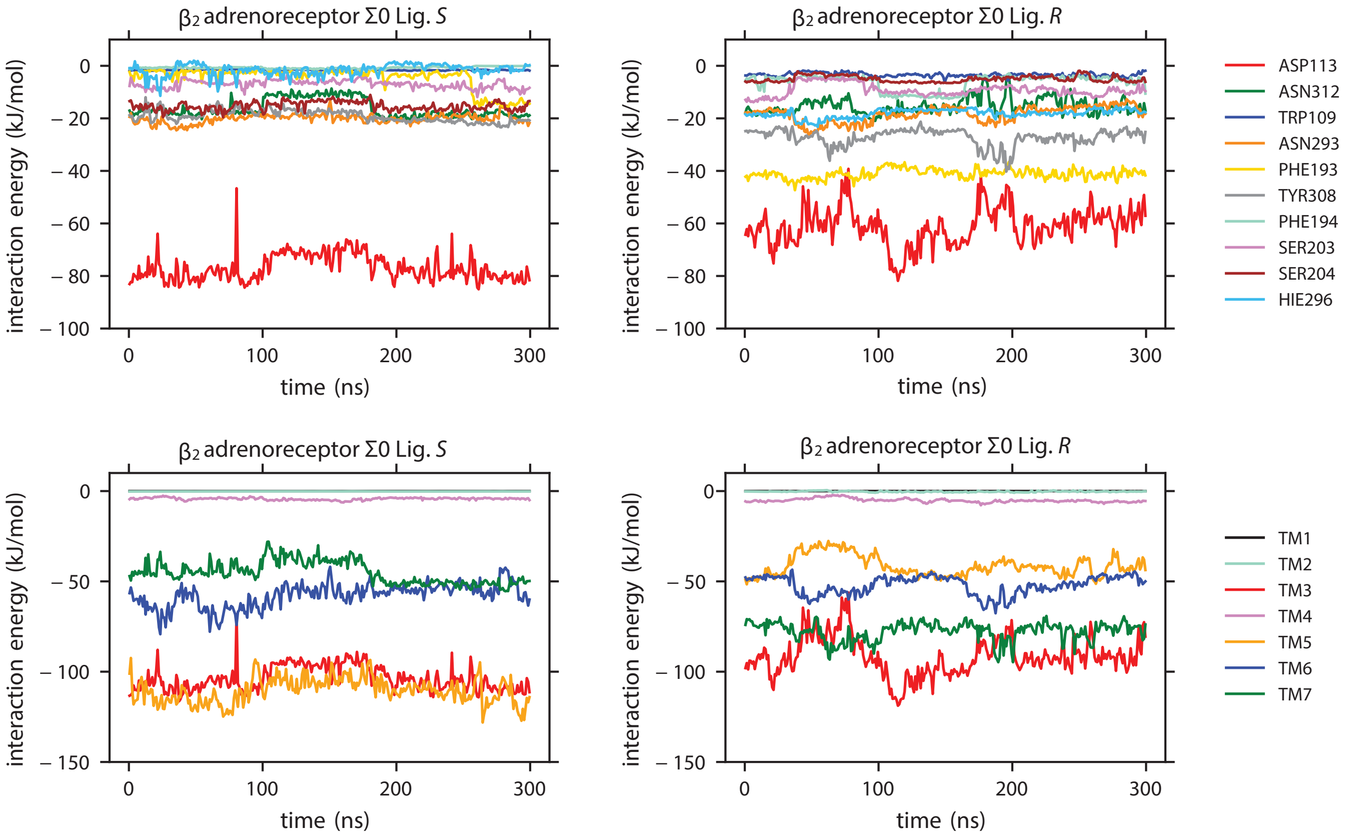

Fig. S13. Time evolution for interaction energies of various β_2 AR residues in the inactive state with the C1-*S* and C1-*R* compounds. The two upper plots show the interaction energies of the ligand (left –C1-*S*, right –C1-*R*) with 10 receptor residues within 5 Å of the ligand. The two lower plots show the interaction energies between the ligand (left –C1-*S*, right –C1-*R*) with the 7 TM domains of β_2AR in the inactive state. The key difference in the interactions is in the aromatic bonds. In the upper plots, interactions with Phe193^{ECL2} (yellow) and Tyr308$^{7.35}$ (grey) are stronger for C1-*R* than for C1-*S*. In addition, Ser203$^{5.42}$ (pink), Phe193^{ECL2} (cyan) and His296$^{6.58}$ (light blue) represent stronger binding of C1-*R* compared to C1-*S*. The most significant contributions to the binding are from the aromatic rings in the extracellular domain, making it difficult to capture in the lower plots where only TM interactions are included.

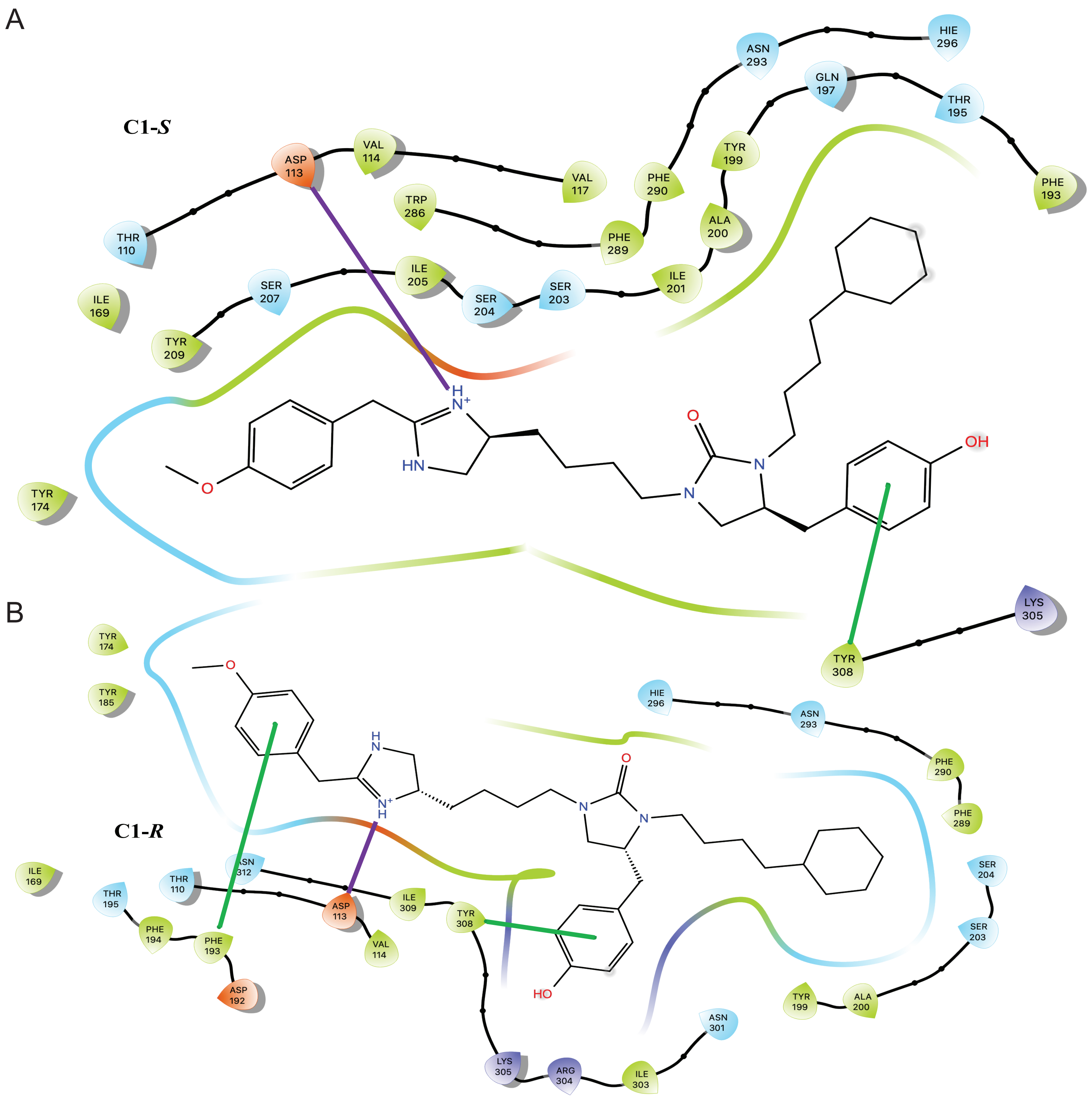

Fig. S14. Comparison of pharmacophores for C1-*S* and C1-*R* interacting with inactive β_2AR. (A) C1-*S* binding site: a SB (purple line) to Asp113$^{3.32}$, and Pi-Pi stacking with Tyr308$^{7.35}$. (B)The binding site of C1-*R* is characterized by the SB to Asp113$^{3.32}$ and two stable aromatic bonds to Phe193^{ECL2} and Tyr308$^{7.35}$ (or His296$^{6.58}$), which are absent or stronger than those formed by C1-*S* (see energy plots in *SI Appendix*, Fig. S13).

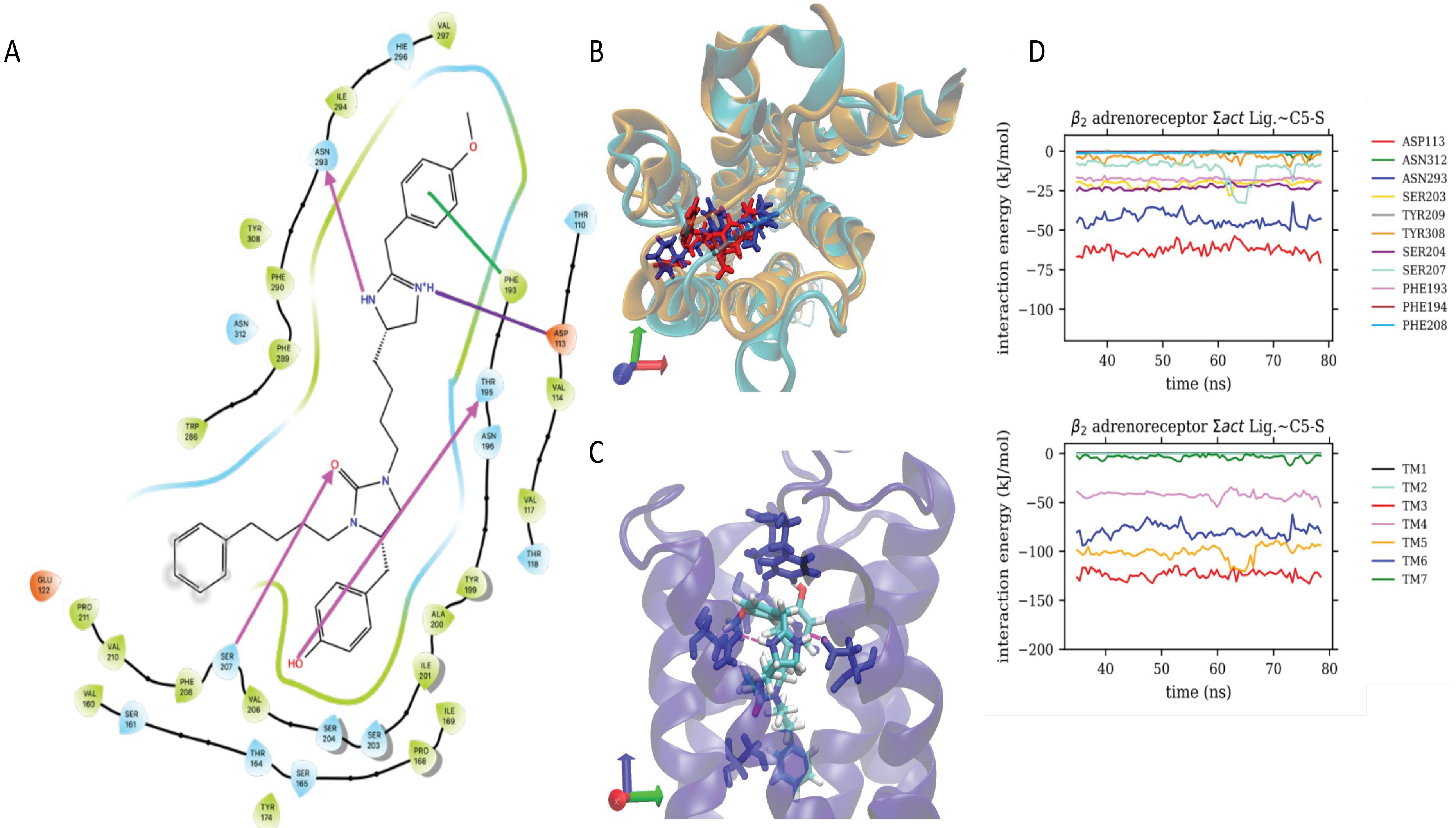

Fig. S15. Predicted binding site for C5-*S* binding to β_2AR coupled to Gs in explicit membrane and water. (A) pharmacophore for predicted binding sites of C5-*S* ligand bound to active-state β_2AR. The C5-*S* binding site includes HB (pink arrows) to Ser203, Asn293, and Thr195, salt bridge (SB, purple line) to Asp113; Pi-stacking at Phe193 with an internal aromatic bond (green line). Ligand atoms that are exposed to solvent are marked with gray spheres. (B) Upper view of C5-*S* (red) in the TM3-4-5-6 pocket in comparison to C1-*S* (blue). (C) The imidazole subunit of C5-*S* forms a salt bridge to Asp113 and an H-bond with Asn293. (D) Time evolution for interaction energies of various β_2AR residues in the inactive state with C5-*S*. The upper plot shows the interaction energies of the ligand with 11 receptor residues within 5 Å of the ligand. The lower plot shows the interaction energies between the ligand with the 7 TM domains of β_2AR in the active state.

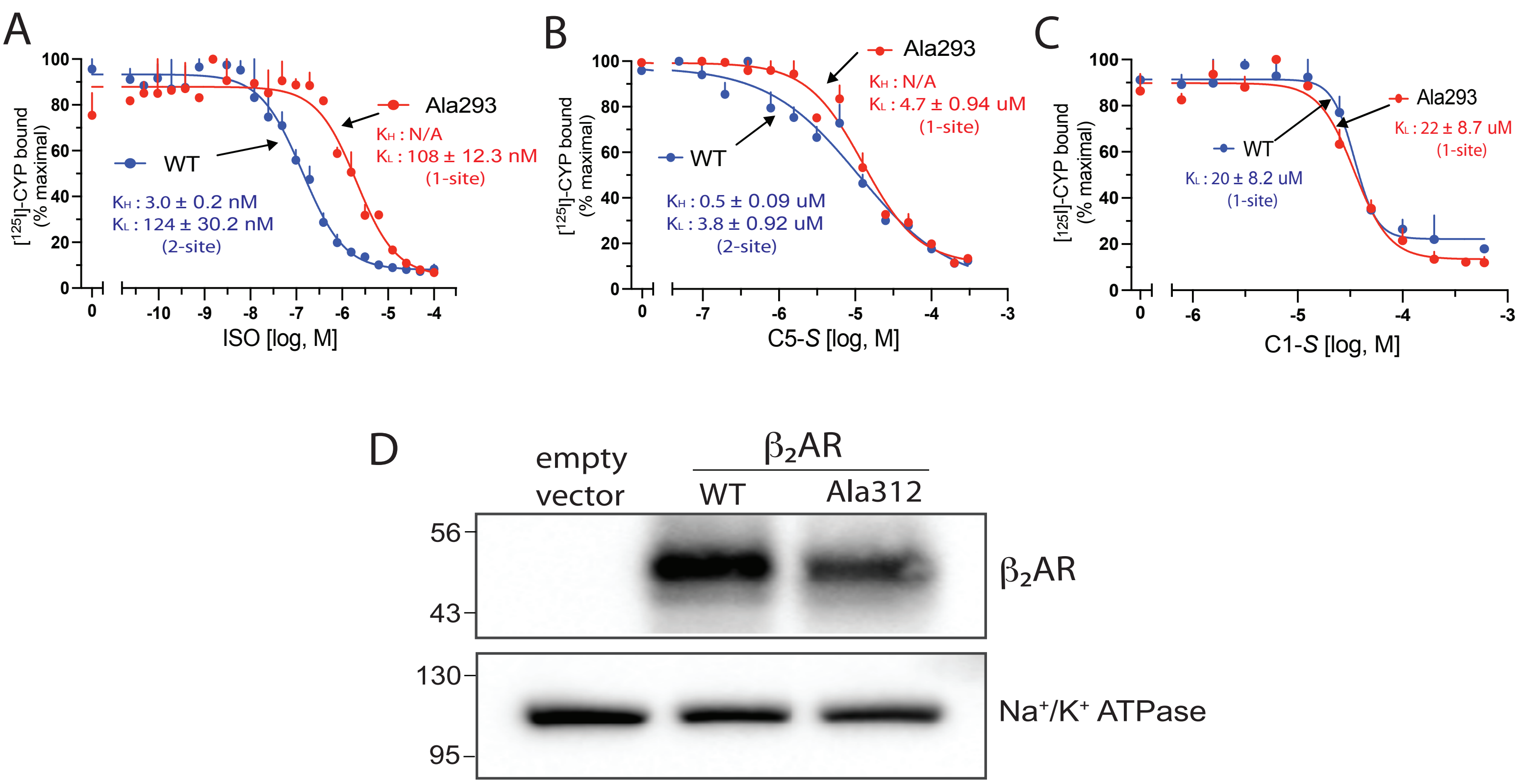

	empty vector	WT	Ala312
β_2AR/ATPase (Ratio)	0	1.963	1.457
[^{125}I]-CYP (400 pM) specific binding, fmol/mg	4 ± 1.8	576 ± 15.9	27 ± 2.6
[^{3}H]-DHA (5 nM) specific binding, fmol/mg		321 ± 17.2	9.9 ± 6.7

Fig. S16. Radioligand agonist competition binding with the β_2AR WT and the Ala293 mutant. HEK-293T cells were transiently transfected with the indicated expression constructs. (A-C) Competition binding studies performed with washed membranes in the absence of GTP. The Ala293 substitution resulted in a loss of high-affinity binding for ISO and C5*S* . The C1*S* competition studies did not show a significant high-affinity site with WT (or the mutant) and these curves were essentially superimposable. The figures show representative curves. The data shown within each figure are mean ± SE of 4-6 experiments each performed in quadruplicate. (D) The Ala312 mutant failed to bind two radioligands, but was expressed on the cell surface. A representative western blot using a polyclonal β_2AR antibody reveals similar expression of WT and Ala312 with cell membrane preparations. The β_2AR signal was normalized to the signal from Na^+/K^+ ATPase (exclusively expressed on the cell surface). ^{125}I-CYP radioligand binding with the indicated saturating concentration of radioligand revealed specific binding for WT at the expected levels, while Ala312 revealed little specific binding compared to the membrane expression observed from western blots. In separate experiments, ^{3}H-DHA radioligand binding using a saturating concentration mirrored those using ^{125}I-CYP. Cells in 10 cm^2 dishes were transfected with 20 ug each of the respective constructs and equal amounts of protein were loaded on the electrophoresis gel. Based on data in (D) radioligand competition studies could not be performed with Ala312.

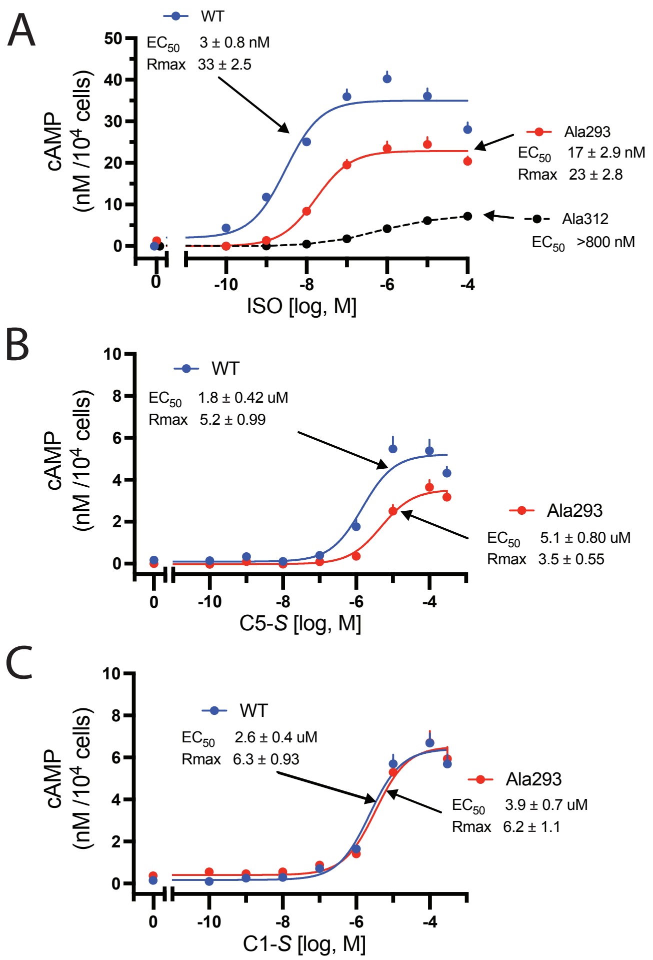

Fig. S17. Functional signaling to cAMP accumulation with β_2AR WT and mutant receptors. (A) Responses to ISO revealed impaired signaling of the Ala293 mutant with a decrease in the Rmax and an increase in the EC_{50} compared to WT. The Ala312 receptor stimulation with the full agonist ISO was decreased by >80% and the EC_{50} increased by ~2-logs. Taken together with the lack of antagonist radioligand binding to Ala312 (see Fig. S16), we conclude that the binding pocket is markedly distorted in this mutant and cAMP studies with the partial agonists C1-*S* and C5-*S* were not performed with Ala312. (B,C) Ala293 responses to C5-*S* were impaired with the mutant but the responses to C1-*S* were not. Data shown are mean ± SE from 3-5 independent experiments performed in quadruplicate.

SI Expanded Methods

Bias Factor Calculations

The bias factors (β) were calculated based on the equiactive method (1, 2) which utilizes the maximal response (Emax = Rmax – Rmin) and the EC_{50} derived from logistic curve fits from dose-response experiments for the two pathways of interest to calculate the activity ratios. Gs activation was determined by the BRET2 assay and β–arrestin (arr) binding by the ECA. The reference agonist is isoproterenol (ISO) and the equation for calculating the bias factor β is thus:

$$\beta = \log\left(\left(\frac{E_{max,Gs}}{EC_{50,Gs}}\frac{EC_{50,arr}}{E_{max,arr}}\right)_{agonist} * \left(\frac{E_{max,arr}}{EC_{50,arr}}\frac{EC_{50,Gs}}{E_{max,Gs}}\right)_{ISO}\right)$$

Dose-response curves for each pathway were fit to a 3-parameter iterative non-linear least squares logistic regression equation with Hill coefficient of 1.0, using Prism 9 (GraphPad, San Diego, CA). Any attempted fit of the data from either pathway that failed to converge with 10,000 iterations or had an R^2 value of <0.75 were not included in the calculation of β. The error for β was calculated using methods similar to those of Costa and colleagues (2). Using the above methods and the indicated formula, the full agonist ISO has a β=0 since it represents a balanced agonist as the reference. Thus, an agonist biased *towards* β-arrestin compared to Gs has β<0, and those with β>0 are biased in favor of Gs compared to β-arrestin. If a signal from one pathway could not be detected (statistically not different than non-agonist control values), the formal β is considered undefined. Under these circumstances, the direction of the apparent bias can be ascertained by inspection of the two responses, and is indicated in the text.

Modeling Methods

We started with the crystal structure (PDB ID: 2RH1) (3) to model the inactive state of the human β_2-adrenergic receptor (β_2AR). In this crystal structure, the ICL3 was replaced with the fusion T4 lysozyme protein. Thus, we eliminated T4 lysozyme from the structure and added an extended conformation of native ICL3, subsequently refined by the MODELLER program (4). Additionally, we also modeled palmitoyl-cysteine 341 in the protein construct.

To model the active state of β_2AR, we started with the crystallographic fully activated β_2AR-bound to BI167107 and coupled with the nucleotide-free Gs protein (PDB ID: 3SN6) (5). We removed the fusion T4 lysozyme along with a co-crystallized nanobody from the construct. Additionally, we built in 24 missing residues (sequence between ^{240}D-N^{264}) of ICL3, and 3 missing residues (^{176}A-H^{178}) of extracellular loop 2 to the β_2AR and refined them with MODELLER (4). During the calculations, we also included the palmitoyl-cysteine 341 in the β_2AR construct. To model the heterotrimeric Gs protein, we used the short isoform of Gs protein, which is the crystallized Gs protein (5). To add palmitoyl-cysteine 3 in the Gαs subunit, we built the first 9 residues of the Gαs-αN helix in the construct of the Gs protein.

We studied C1-S and C1-R, identical compounds but with opposite chirality at the R1 position, and C5-S. To predict the binding site of this large ligand, we first used the Maestro software (Schrödinger, Inc) to divide each compound into 3 fragments (SI Appendix, Fig. S10): the imidazole moiety, the urea moiety, and the cyclohexane alkyl chain; and LigPrep to generate accurate, energy minimized 3D molecular structures of these fragments. We applied Conformational Searching to find all low energy conformations for each molecule. We used quantum mechanics (QM) methods in Jaguar (Schrödinger, Inc) to optimize the geometry and to predict the Mullikan charges (6). Particularly, we used the Becke, three-parameter, Lee-Yang-

Parr (B3LYP) (7, 8) and the 6-31G** basis set (9, 10), and included the van der Waals corrections.

We applied the DarwinDock complete sampling method to predict the binding sites and energetics of the three fragments as described previously (11-14). This involved replacing the 6 hydrophobic residues of β_2AR with alanine to provide space for ligand docking. We then generated ~50,000 poses sufficient to span the putative binding regions of the alanized protein. To ensure complete sampling, these poses were generated in increments of 5,000 and clustered into Voronoi families based on root mean square deviation (RMSD) until <2% new families were generated. The family heads were energy scored using the Dreiding force field with the top 10% selected by interaction energy. Then interaction energies for all children of these top 10% families were calculated to finally select the lowest energy 100 poses for further optimization. The receptor was then dealanized (replacing the Ala for the 6 hydrophobic residues with optimum side chains) using the side-chain rotamer excitation analysis method (SCREAM) (15) for each of the 100 poses. To avoid effects of long-range Coulomb interactions on the binding energies, we neutralized the protein and the ligand by transferring protons appropriately within salt bridges and protonating or deprotonating exterior ligands, followed by further full geometry minimization. We then compared the energies for the 100 structures to select ~5 with the best binding energies. This was done for each of the 3 fragments. We then combined them to determine binding poses for the full compound. For subsequent studies we used the full compound C1-*S*, C1-*R*, or C5-S with the β_2AR alone or in complex the G protein α5 subunit. Then we equilibrated (annealed) in a vacuum from a starting temperature of 25º K up to the final temperature of 600º K over 1 ns using GROMACS (Uppsala University, Sweden) with the Amber force field (http://ambermd.org/AmberModels.php). Then we equilibrated in a vacuum for 100 ns at 310 º K to find the best position for the highly flexible cyclohexane alkyl fragment.

Next, we inserted the ligand-GPCR-G Protein complex into a preequilibrated palmitoyl-oleoyl-phosphatidylcholine (POPC) bilayer structure by superimposing b2AR to the k-opiod receptor pre-equilibrated in the POPC membrane bilayer which was composed of 277 POPC molecules (11). We used GROMACS to place the membrane and protein in a 100 × 100 × 170 Å cubic box (~39,000 water molecules) with the extracellular and intracellular regions (above and below the POPC membrane) filled with a 298º K preequilibrated water (defined by the TIP3P model (16)), followed by addition of ions (final NaCl concentration of 100 mM). The protein was described using Amber14 while parameters for the POPC were taken from LIPID17 in Ambertools (https://www.advancedhpc.com/pages/amber). The carboxymethylation of the lipid linkages were described by the parameters obtained from the generalized Amber force field using ACPYPE (17) and Antechamber16 (http://ambermd.org/antechamber/ac.html). The partial charges for these two were assigned with the semi-empirical AM1-BCC model, which is incorporated in UCSF Chimera (18).

Molecular Dynamics (MD) simulations

Simulations were carried out using GROMACS. To optimize each ligand-β_2AR-Gs complex, we carried out 5000 steps of energy minimization using the steepest descent algorithm. Subsequently, we carried out a ~1ns MD simulation where we placed positional restraints on the heavy atoms of proteins, and ligands with a force constant of 9.6 kcal.mol^{-1} Å^{-2}. In addition, we restrained the z-coordinate of the headgroups of POPC inside the membrane with a force constant of ~2.4 kcal/mol/Å so that the POPC molecules can move freely along the xy-plane to find their appropriate packing. Throughout these calculations, the restraints on the proteins, ligands, and POPC were gradually reduced to 0 kcal/mol/Å, which prepared the construct for the further relaxation. Then, we removed all restraints and performed ~1 μs MD simulation in an isothermal-isobaric ensemble to relax the complex. The last 100ns was used for analysis. The

temperature was maintained at 310º K using a velocity-rescale (19) thermostat with a damping constant of 1.0 ps for temperature coupling and the pressure was controlled at 1 bar using a Parrinello-Rahman barostat algorithm with a 5.0 ps damping constant for the pressure coupling. Semi-isotropic pressure coupling was used during this calculation. The Lennard-Jones cutoff radius was 12 Å, where the interaction was smoothly shifted to 0 after 10 Å. Periodic boundary conditions were applied to all three directions. The Particle Mesh Ewald algorithm with a real cutoff radius of 10 Å and a grid spacing of 1.2 Å was used to calculate the long-range coulombic interactions. Compressibility of 4.5 x 10^{-5} bar^{-1} was used in the xy-plane and also the z axis, to relax the box volume. In all the above simulations, water OH-bonds were constrained by the SETTLE algorithm. The remaining H-bonds were constrained using the P-LINCS algorithm.

1. S. Rajagopal *et al.*, Quantifying ligand bias at seven-transmembrane receptors. *Mol Pharmacol* **80**, 367-377 (2011).
2. H. O. Onaran *et al.*, Systematic errors in detecting biased agonism: Analysis of current methods and development of a new model-free approach. *Scientific reports* **7**, 44247 (2017).
3. V. Cherezov *et al.*, High-resolution crystal structure of an engineered human beta2-adrenergic G protein-coupled receptor. *Science* **318**, 1258-1265 (2007).
4. N. Eswar, D. Eramian, B. Webb, M. Y. Shen, A. Sali, Protein structure modeling with MODELLER. *Methods Mol Biol* **426**, 145-159 (2008).
5. S. G. Rasmussen *et al.*, Crystal structure of the β2 adrenergic receptor-Gs protein complex. *Nature* **477**, 549-555 (2011).
6. A. D. Bochevarov *et al.*, Jaguar: A high-performance quantum chemistry software program with strengths in life and materials sciences. *Int J Quantum Chem* **113**, 2110-2142 (2013).
7. A. D. Becke, Density-Functional Thermochemistry .3. The Role of Exact Exchange. *J Chem Phys* **98**, 5648-5652 (1993).
8. C. Lee, W. Yang, R. G. Parr, Development of the Colle-Salvetti correlation-energy formula into a functional of the electron density. *Phys Rev B Condens Matter* **37**, 785-789 (1988).
9. R. Ditchfield, W. J. Hehre, J. A. Pople, Self-Consistent Molecular-Orbital Methods .9. Extended Gaussian-Type Basis for Molecular-Orbital Studies of Organic Molecules. *J Chem Phys* **54**, 724-728 (1971).
10. W. J. Hehre, J. A. Pople, Self-Consistent Molecular-Orbital Methods .13. Extended Gaussian-Type Basis for Boron. *J Chem Phys* **56**, 4233-4234 (1972).
11. A. Mafi, S. K. Kim, W. A. Goddard, 3rd, The atomistic level structure for the activated human kappa-opioid receptor bound to the full Gi protein and the MP1104 agonist. *Proc Natl Acad Sci U S A* **117**, 5836-5843 (2020).
12. A. Griffith (2017) DarwinDock and GAG-Dock: Methods and Applications for Small Molecule Docking. in *Chemistry and Chemical Engineering* (California Institute of Technology, Pasadena, CA), p 171.
13. S. K. Kim, L. Riley, R. Abrol, K. A. Jacobson, W. A. Goddard, 3rd, Predicted structures of agonist and antagonist bound complexes of adenosine A3 receptor. *Proteins* **79**, 1878-1897 (2011).
14. W. A. Goddard, 3rd *et al.*, Predicted 3D structures for adenosine receptors bound to ligands: comparison to the crystal structure. *J Struct Biol* **170**, 10-20 (2010).
15. V. W. Tak Kam, W. A. Goddard, 3rd, Flat-Bottom Strategy for Improved Accuracy in Protein Side-Chain Placements. *J Chem Theory Comput* **4**, 2160-2169 (2008).
16. P. Mark, L. Nilsson, Structure and dynamics of the TIP3P, SPC, and SPC/E water models at 298 K. *J Phys Chem A* **105**, 9954-9960 (2001).
17. A. W. Sousa da Silva, W. F. Vranken, ACPYPE - AnteChamber PYthon Parser interfacE. *BMC Res Notes* **5**, 367 (2012).
18. E. F. Pettersen *et al.*, UCSF Chimera--a visualization system for exploratory research and analysis. *J Comput Chem* **25**, 1605-1612 (2004).
19. G. Bussi, D. Donadio, M. Parrinello, Canonical sampling through velocity rescaling. *J Chem Phys* **126**, 014101 (2007).
